# A facile method for fluorescent visualization of newly synthesized fibrous collagen by capturing the allysine aldehyde groups serving as cross-link precursors

**DOI:** 10.1101/2025.06.19.660320

**Published:** 2025-06-24

**Authors:** Junpei Kuroda, Kazunori K. Fujii, Sugiko Futaki, Azumi Hirata, Yuki Taga, Takaki Koide

**Affiliations:** 1 Laboratory of Morphogenesis, JT Biohistory Research Hall, Takatsuki, Osaka, Japan.; 2 Graduate School of Science, Osaka University, Toyonaka, Osaka,Japan.; 3 Graduate School of Frontier Bioscience, Osaka University, Suita, Osaka,Japan.; 4 Department of Chemistry and Biochemistry, Waseda University, Shinjuku-ku, Tokyo, Japan; 5 Department of Anatomy and Cell Biology, Osaka Medical and Pharmaceutical University, Takatsuki, Osaka, Japan.; 6 Nippi Research Institute of Biomatrix, Toride, Ibaraki, Japan; 7 Waseda Research Institute for Science and Engineering, Waseda University, Shinjuku-ku, Tokyo, Japan.

## Abstract

The fibrous structures of collagen provide physical strength and stability to tissues and organs. Abnormalities in their orientation, growth, and remodeling cause morphogenetic defects and serious diseases including fibrosis, so it is important to clarify how collagen fibers are correctly oriented and grown within tissues. However, this mechanism remains elusive, as few methods have been available to fluorescently stain collagen fibers with a simple protocol and to observe their structure in three dimensions. Here we present a facile method that enables fluorescent staining of collagen fibers in vertebrate tissues. In our method using DAF-FM, known as a NO detection probe, premature collagen fibers can be visualized via covalent binding to the allysine residues serving as precursors of cross-linking structures of collagen. In addition, we showed that the labeling method using two fluorescent probes with different colors, DAF-FM and DAR-4M, allows for pulse-chase observation of newly synthesized collagen fibers. Our method will be a breakthrough technique in future collagen studies.

## INTRODUCION

The optimal function and physical properties of tissues are highly dependent on the orientation pattern of the supramolecular structure of extracellular matrix (ECM) and its density (1, 2). Collagen is known as a core ECM protein that forms fibers, and it not only provides physical strength to the tissues but also serves as a scaffold for cells to maintain tissue function and homeostasis (1–6). In the morphogenesis of various tissues such as bone, tendon, skin, and cornea, regularly assembled fibrous collagen contributes to normal shaping as a building material for supporting tissues (2, 5). During the process of bone formation, abnormalities of collagen fibers in the orientation and in the density due to errors in formation of suprastructure lead to a bone disease, osteogenesis imperfecta (7, 8). In addition, abnormal production of collagen fibers and disruption of their remodeling processes trigger fibrosis in various tissues (9, 10). In order to clarify the mechanisms of the pathogenesis of these diseases, it is necessary to understand the dynamics of collagen fibers distributed in tissues. Therefore, it is essential to develop techniques to easily visualize collagen architecture in living tissues, as well as a new imaging tool to track the dynamics of collagen fiber growth.

To visualize and analyze collagen fibers, several techniques have been developed. Among these, fluorescent staining and labeling are powerful tools to obtain information about the shape and spatial distribution of collagen fibers within tissues. Immunofluorescent staining is a common method to observe the distribution of these fibers in various studies (11–13). Despite recent advancements in tissue transparency technologies that facilitate deep tissue immunofluorescent staining, achieving high-clarity images with minimal background noise and nonspecific signals remains a significant challenge. SHG imaging with multiphoton microscopy is a noninvasive technique to visualize collagen fibers in various tissues without fixation or staining (14, 15). However, this method is not suitable for observing at early morphogenesis stages such as embryogenesis, as it is difficult to detect sufficient second-harmonic generation (SHG) signal in immature fine fibers. Alternatively, recent studies have achieved *in vivo* fluorescence imaging of fibrillar collagen using GFP labeling (16–19). This technique is effective for live imaging, similar to SHG imaging, and it also allows the visualization of fine fibers that are hard to see with SHG imaging. Despite these advantages, this observation system requires a great deal of effort to introduce gene expression constructs and is only applicable to visualization of fibrillar collagen composed of type I collagen. In addition to this, there is concern that in many cases, labeling of collagen molecules with fluorescent proteins such as GFP may interfere with normal folding of collagen molecules and normal fibrogenesis of collagen. Recently, it has been reported that the networks of collagen fibers in various tissues are clearly stained with a synthetic colorant, Fast Green FCF (20). Although this method with low molecular weight chemicals allows visualization of collagen fibers in very deep regions of tissues, specific staining is prevented under hydrophilic conditions, and furthermore, it requires many steps to prepare the sample for staining. Furthermore, new visualization techniques targeting the precursors of cross-linking structures of collagen have recently been reported (21–25), but there is still no rapid and easy method for fluorescent staining of collagen fibers in various vertebrate tissues. In addition, the development of pulse-chase analysis, which allows for the fluorescent labeling of collagen fibers in living tissues and the subsequent tracking of their growth process, could be a breakthrough imaging tool to understand collagen dynamics during tissue growth.

Here, we provide a novel fluorescence imaging method for collagen fibers using diaminofluorescein-FM (DAF-FM), which is widely used as a detection probe for nitric oxide (NO) produced by cells, and its analogs (26–28). We focused on the previous studies that reported DAF-FM DA (diacetate) fluorescently stains the notochord, cartilage, bone and actinotrichia, which are structures of collagen fibers oriented at the tip of zebrafish fin (29–33). Recently, Ohashi et al. reported that this probe fluorescently labeled collagen fibers in the axolotl skin (34). Based on the results of these studies, we hypothesized that this probe could interact with collagen fibers and fluorescently stain them, apart from NO. In this study, we found that DAF-FM fluorescently labels the collagen fibers by a rapid and simple method while the mouse fibroblasts are alive *in vitro*. We also showed that pulse-chase analysis of collagen fibers formed in cell culture condition can be performed by labeling them with DAF-FM and another probe, diaminorhodamine-4M (DAR-4M), at different time points. Using the collagen fibers produced by culture cells, our mass spectrometric analysis successfully revealed that DAF-FM reacted to the aldehyde groups of allysine serving as the precursors of interchain cross-link. Furthermore, we found that collagen fibers, which are oriented deep within mouse tissues, can be clearly and three-dimensionally stained using DAF-FM DA. In addition, we also found that *in vivo* imaging of the collagen fibers using DAF-FM DA can be performed in other vertebrates including zebrafish and axolotl. Finally, we demonstrated that the pulse-chase observation of collagen fibers with these two fluorescent probes can be applied to *in vivo* imaging of tissues such as zebrafish bones. Our method is an innovative imaging technique that targets the cross-linking precursors of collagen fibers, and its rapid and easy method will become a standard tool in future collagen research.

## RESULTS

### DAF-FM and DAR-4M enable fluorescent labeling of collagen fibers formed by cultured cells

**Fig. 1.**
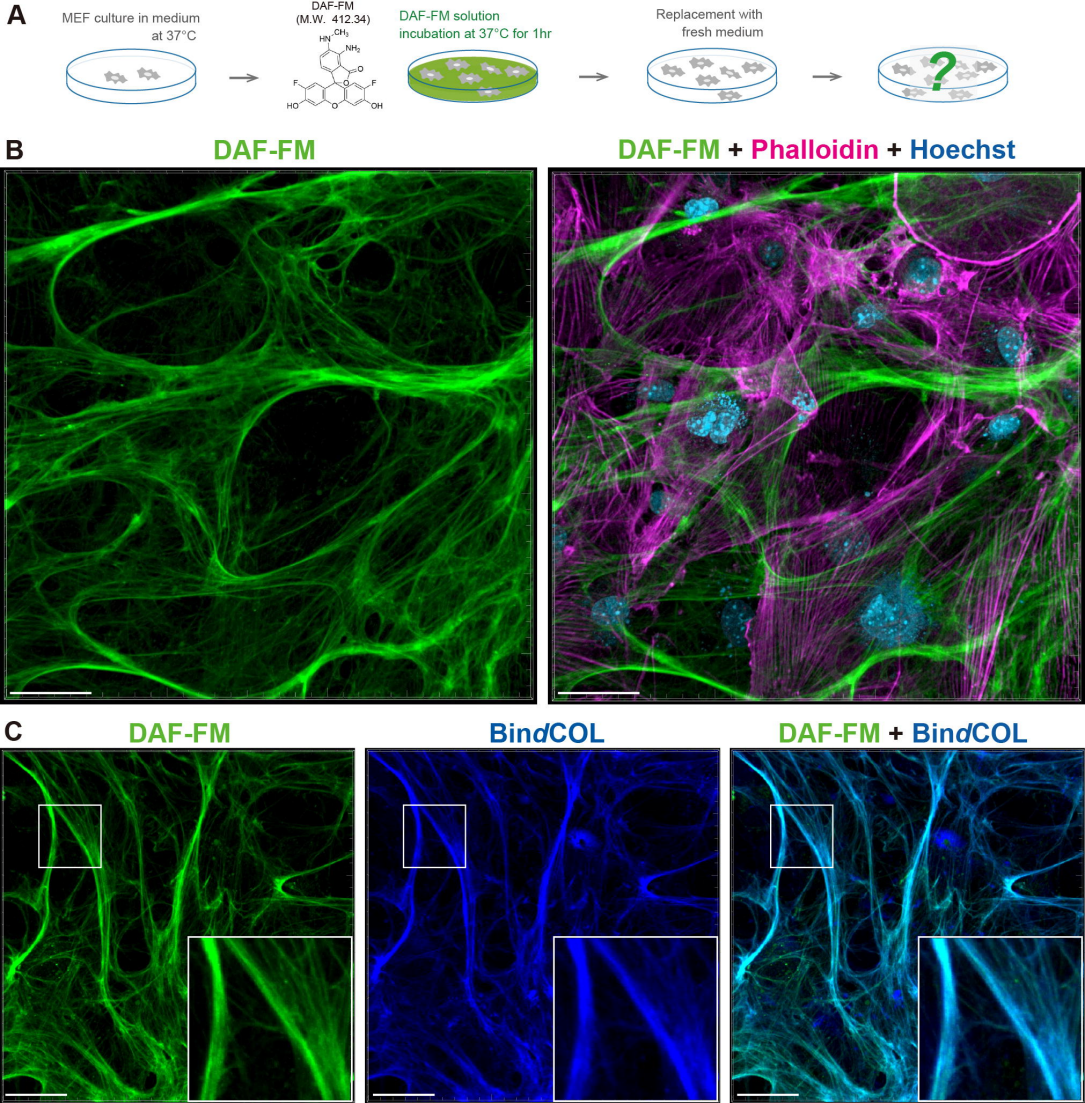
A rapid and simple method using DAF-FM for visualization of the collagen fibers formed by culture cells. (**A**) Schematic diagram of DAF-FM staining for the collagen fibers formed by MEFs. Fully proliferated MEFs after 1 to 2 weeks of culture were incubated in 10μM DAF-FM solution for 1hr. After the incubation, DAF-FM solution was replaced with fresh medium and the collagen fibers visualized by DAF-FM were observed. (**B**) Representative fluorescent images of the collagen fibers labeled by DAF-FM (green) at culture day 10. Actin cytoskeleton was stained with Phalloidin (magenta), and nucleus was stained with Hoechst (blue). (**C**) Representative fluorescent images of the collagen fibers co-stained with DAF-FM (green) and Bin*d*COL (blue). Scale bar = 50 μm.

Previous studies have reported that DAF-FM DA, a known NO detection probe, is effective for fluorescent staining of collagen fiber-rich tissues such as notochord, cartilage, bone, and skin (29–34). Based on the results of these studies, we hypothesized that this probe could fluorescently stain collagen fibers independently of NO. In this research, we first decided to test this possibility by culturing mouse fibroblasts, which have a high activity for the production of collagen fibers. In this experiment, we used DAF-FM, a membrane impermeable version of DAF-FM DA. Before starting the culture experiment, we coated culture dishes with purified collagen to check if these substrates would be fluorescently stained by DAF-FM. We then confirmed that DAF-FM reacts little with purified collagen used as culture substrates (fig. S1). Therefore, we seeded mouse embryonic fibroblasts (MEFs) on gelatin-coated culture dishes and stained them with DAF-FM while the cells were alive after culture (Fig. 1A). As a result, we found that the network of fiber structures formed by MEFs was fluorescently labeled by DAF-FM (Fig. 1B). To determine whether these fibrous structures were collagen fibers, we conducted double staining with DAF-FM and Bin*d*COL, a cyclic peptide that hybridizes to denatured portions of collagen (35). Importantly, fibers labeled by DAF-FM were also simultaneously labeled by Bin*d*COL after denaturation by heat treatment, indicating that DAF-FM fluorescently stains collagen fibers formed by the cultured fibroblasts (Fig. 1B). We also found that when co-stained with DAF-FM and its derivative, DAR-4M, the two probes fluorescently label the same fiber structures (fig. S2). We also performed double staining with DAR-4M using an antibody to type I collagen (Col1) and found that the DAR-4M staining merged very well with the Col1 antibody staining (fig. S2). These results indicate that DAF-FM and DAR-4M can fluorescently label collagen fibers produced by fibroblasts under culture conditions using a simple method.

We next asked whether the fluorescence of collagen fibers observed after DAF-FM staining was due to the action of NO produced by the cells. Therefore, to clarify the involvement of NO, we treated MEFs under the culture conditions to remove NO and observed the fluorescence of fibers after DAF-FM staining. As a result, no significant difference was observed in the intensity of the fluorescent signal from the fibers under NO removal conditions compared to control (fig. S3), indicating that NO is not involved in the fluorescence emission of collagen fibers by DAF-FM.

We also investigated the effect of DAF-FM staining on the cell activities. We treated MEFs in the culture condition with DAF-FM and then performed a BrdU incorporation assay to assess the activity of cell proliferation (fig. S4). As a result, there was no significant difference in BrdU incorporation between cells treated with DAF-FM and untreated cells (fig. S4). In addition, to determine the effect of DAF-FM treatment on cell survival, we checked whether apoptosis was enhanced after DAF-FM treatment. We used nuclear staining reagents to detect apoptosis and found no significant change in cell death between cells treated with DAF-FM and untreated cells (fig. S4). Based on these results, we conclude that DAF-FM does not adversely affect cell activities, at least at low concentrations of treatment, and that this probe allows the visualization of collagen fibers produced by living cells independently of NO.

### Fluorescent labeling of collagen fibers with DAF-FM via interaction with cross-linking intermediate structures

**Fig. 2.**
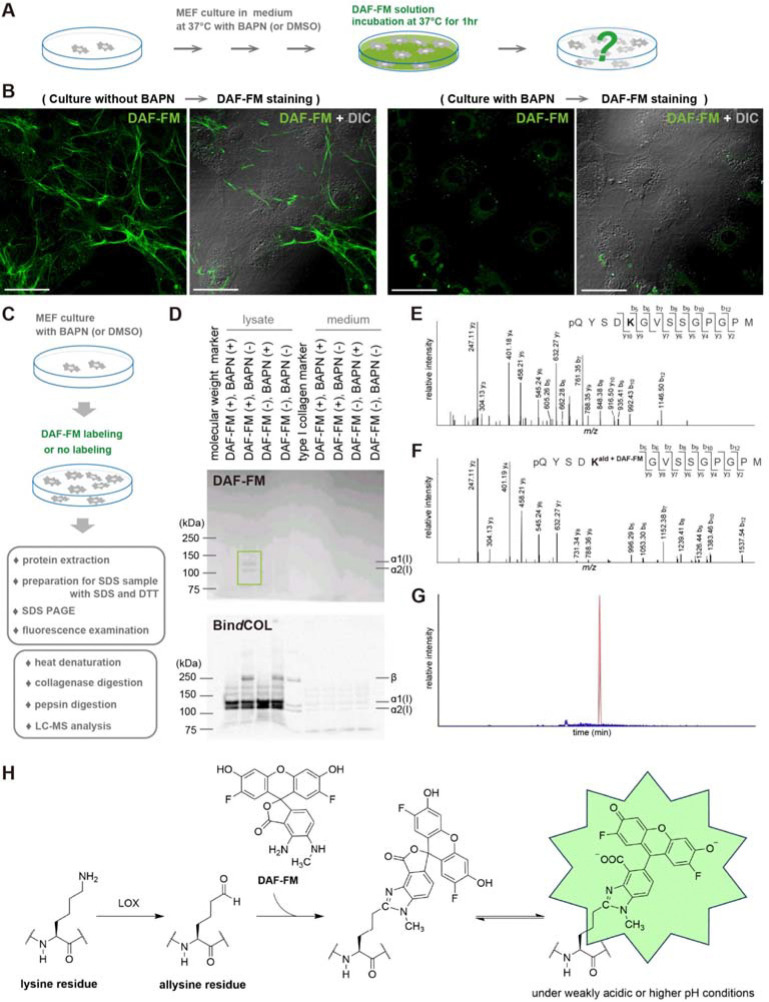
Mechanism of DAF-FM fluorescence targeting collagen cross-linking. (**A**) Schematic diagram of DAF-FM staining for the collagen fibers formed by MEFs under the culture condition with BAPN. (**B**) Representative fluorescent images of the collagen fibers labeled by DAF-FM (green) under the culture condition without BAPN and with BAPN. Scale bar = 50 μm. (**C**) Experimental workflow for sample preparation for SDS-PAGE and LC-MS analysis. (**D**) SDS-PAGE analysis of DAF-FM-labeled collagen. DAF-FM fluorescence of proteins derived from the lysate and medium at each condition was examined (upper panel), and Bin*d*COL staining of the same protein samples at each condition was performed (lower panel). (**E**) MS/MS spectra of α2-N telopeptide containing lysine (*m*/*z* 696.8085, *z* = 2) derived from control (non-labeled) samples. (**F**) MS/MS spectra of α2-N telopeptide containing DAF-FM-labeled allysine (*m*/*z* 892.3239, *z* = 2) derived from DAF-FM-labeled samples. (**G**) Monoisotopic extracted ion chromatograms of the DAF-FM-labeled α2-N telopeptide (*m*/*z* 892.3352 ± 0.02, *z* = 2) for the control (blue) and DAF-FM-labeled sample (red). (**H**) Proposed mechanism of collagen fiber visualization with DAF-FM.

To clarify the affinity and specificity of DAF-FM for collagen fibers, we attempted to identify the mechanism by which this fluorescent probe labels collagen fibers. DAF-FM is widely used as a probe that reacts with NO to emit fluorescence (36), but the result in fig. S3 indicates that NO is not involved in the fluorescent labeling of collagen fibers by DAF-FM. Here we note that DAF-FM has recently been reported to emit green fluorescence by binding to aldehydes as well as NO (37). In addition, it is known that ε-amino groups of lysine residues in the N- and C-terminal non-triple helical domains (telopeptides) of collagen α-chain are modified to aldehyde groups by lysyl oxidase (LOX), and collagen molecules undergo intra- and intermolecular cross-linking via these aldehyde groups, resulting in the polymerization and growth of collagen fibers (38–40). Based on these facts, DAF-FM was suggested to fluorescently label collagen fibers by interacting with aldehydes, which are intermediate structures in collagen cross-linking. Therefore, we examined the fluorescence after DAF-FM staining under conditions that inhibit the formation of collagen cross-linking. We treated MEFs in culture condition with L-aminopropionitrile (BAPN), known as an inhibitor of LOX (41), in this experiment (Fig. 2A). As a result, inhibition of collagen cross-linking formation by treatment with BAPN suppressed fluorescent labeling of fibers by DAF-FM (Fig. 2B). On the other hand, when MEFs were cultured under normal conditions and then stained with DAF-FM in the presence of BAPN, clear fluorescence of collagen fibers was observed, similar to the control (fig. S5A). These results suggest that the fluorescent labeling of collagen fibers by DAF-FM is closely related to the modification of collagen by LOX.

We next tried to determine the specific target sites of collagen cross-linking structure that DAF-FM interacts with. First, proteins were extracted from MEF culture dishes treated with DAF-FM and SDS-PAGE was performed under reducing condition using DTT to examine whether DAF-FM fluorescence was retained (Fig. 2C). As a result, when MEFs were cultured in the absence of BAPN and stained with DAF-FM, specific bands were detected in the lysate-derived sample at the positions of molecular weights predicted to be α1(L) and α2(L) chains (Fig. 2D). On the other hand, these specific bands were not detected in the lysate-derived sample when the same experiment was performed in the presence of BAPN (Fig. 2D, fig. S5B). We also tested whether the same bands could be detected by Bin*d*COL after the same experiment. In the lysate-derived sample, specific bands labeled with Bin*d*COL were detected at the positions of molecular weights predicted to be α1(L) and α2(L) (Fig. 2D, fig. S5B). These experimental results indicate that under culture conditions in the presence of BAPN, production of collagen fibers itself occurs normally, but the inhibition of cross-linking suppresses fluorescence caused by DAF-FM. Furthermore, the fluorescence caused by DAF-FM in the two specific bands did not disappear after pepsin treatment, indicating that DAF-FM specifically reacts with collagen molecules that are protease-resistant due to their triple helical structure (fig. S5C). In addition, our results also suggest that DAF-FM fluoresce through covalent binding to specific structures that occur during the cross-linking formation process.

We further attempted to identify the binding site of DAF-FM by LC-MS analysis (Fig. 2C). In this experiment, we analyzed three collagenase/pepsin-digested peptides derived from telopeptide domains of type I collagen (α1-N telopeptide, α1-C telopeptide and α2-N telopeptide) which contain lysine or hydroxylysine participating in cross-link formation. The telopeptidyl lysine residues are not hydroxylated in skin (42), while they are largely converted to hydroxylysine in bone (43). Thus, we analyzed lysine-containing telopeptides for the MEF samples. MS/MS sequence analysis confirmed that DAF-FM binds to the aldehyde groups of lysine residues within the α2-N telopeptide in the DAF-FM-labeled sample (Fig. 2E and F). Extracted ion chromatograms showed that a strong peak of this telopeptide labeled with DAF-FM was detected only in the DAF-FM-labeled sample (Fig. 2G). Extracted ion chromatogram peaks corresponding to the theoretical molecular weight of α1-N and α1-C telopeptides attached with DAF-FM were only slightly detected in the DAF-FM-labeled sample, and MS/MS sequence confirmation could not be performed (data not shown). The mass shift observed in the MS/MS spectra suggests that DAF-FM reacted with aldehyde groups to form fluorophores through the same mechanism as previously reported (37). The predicted fluorophore is generated by the reaction of DAF-FM with the aldehyde group of the allysine residue (Fig. 2H). Based on these results, we conclude that DAF-FM fluorescently labels collagen fibers by covalently binding to the aldehyde groups of allysine (and possibly hydroxylysine) residues in the telopeptide, intermediate structures in collagen cross-linking.

### Fluorescent visualization of collagen fibers with DAF-FM DA in cartilage and notochord of mouse embryos

**Fig. 3.**
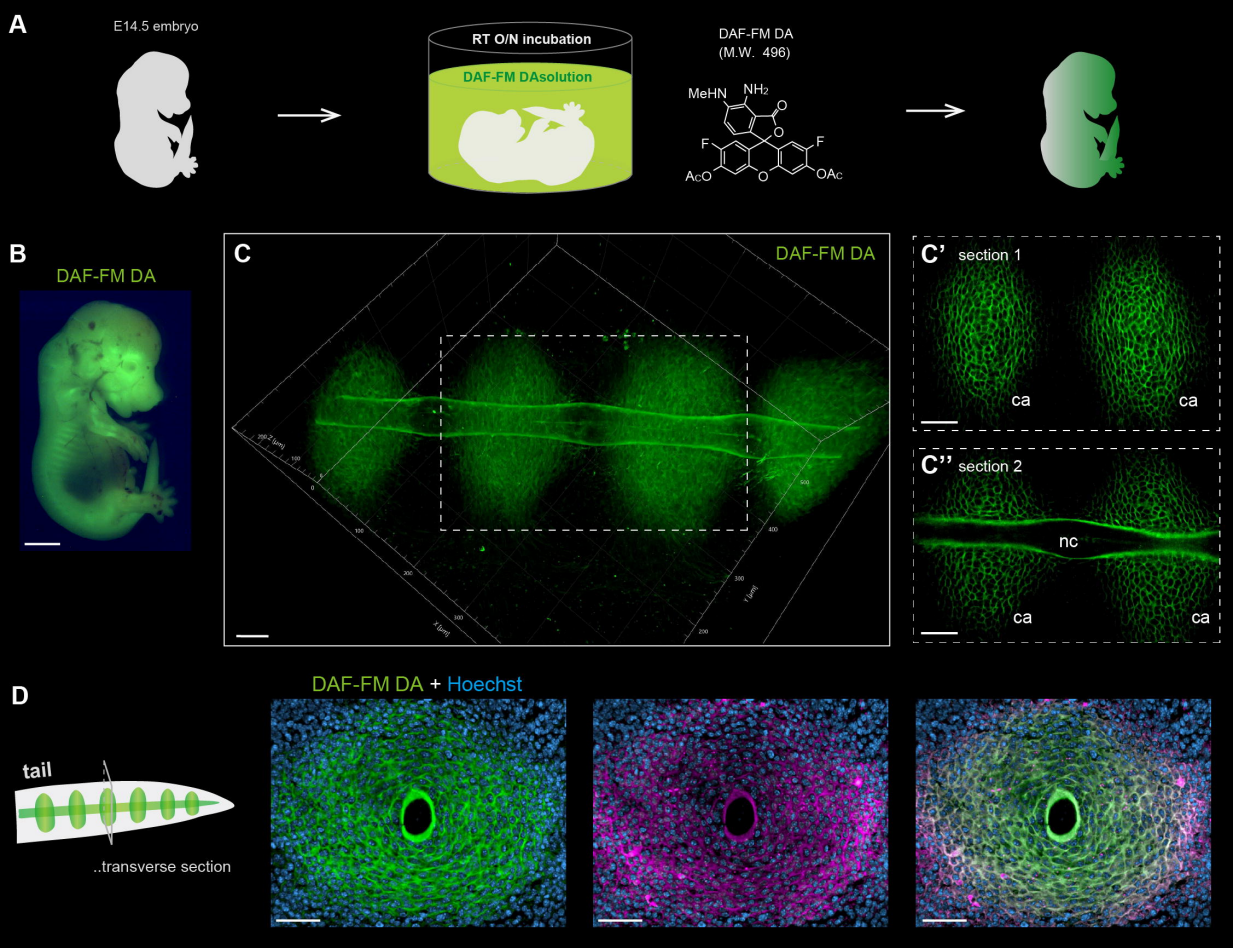
DAF-FM DA enables clear fluorescent visualization of the collagen fibers in cartilage and notochord of mouse embryos with a simple method. (**A**) Schematic diagram of DAF-FM DA staining for the collagen fibers in mouse embryos. (**B**) The fluorescent image of the E14.5 embryo after DAF-FM DA staining. (**C**) The 3D fluorescent image of the embryonic tail stained with DAF-FM DA. Slice images of the area within the white dotted box are shown in (C’ and C”). ca, cartilage; nc, notochord. (**D**) The fluorescent images of antibody staining of the cryo-section samples of the tail. The tissue sections labeled with DAF-FM DA (green) were stained with anti-Col2 antibody (magenta) and Hoechst (blue). Scale bar = 2 mm (B) and 50 μm (C to D).

Using mouse culture cells, we successfully demonstrated that DAF-FM fluorescently labels collagen fibers through a mechanism distinct from that of NO detection. We then tried to DAF-FM staining using mouse tissues to determine if this probe is also useful for fluorescent imaging of collagen fibers *in vivo*. In previous studies, DAF-FM DA, which exhibits plasma membrane permeability (27), was used for whole-body fluorescence staining of zebrafish (29–32). Therefore, we assumed that DAF-FM DA has high tissue penetration and decided to use this probe for fluorescent staining of mouse tissues. We first examined whether two collagen-rich tissues, cartilage and notochord, could be fluorescently stained with DAF-FM DA in mouse embryos. Unfixed embryos at embryonic day (E) 14.5 were incubated overnight in a diluted DAF-FM DA solution (Fig. 3A and B). After incubation, tails of embryos were cleared to examine the three-dimensional orientation of collagen fibers in the cartilage and notochord. Confocal microscopy imaging revealed that strong green fluorescent signals were detected in the cartilage primordium and notochord (Fig. 3C–C”). To determine whether these DAF-FM DA signals were derived from collagen fibers, the sections of DAF-FM DA-labeled embryo were incubated with an anti-type II collagen (Col2) antibody. Reactivity of type II collagen, the major extracellular matrix component of the cartilage, was detected in the area where DAF-FM DA fluorescence was observed (Fig. 3D), indicating that DAF-FM DA also fluorescently labels developing collagen fibers in mouse embryos.

### Fluorescent visualization of collagen fibers with DAF-FM DA in tendons and cartilage of postnatal mouse

**Fig. 4.**
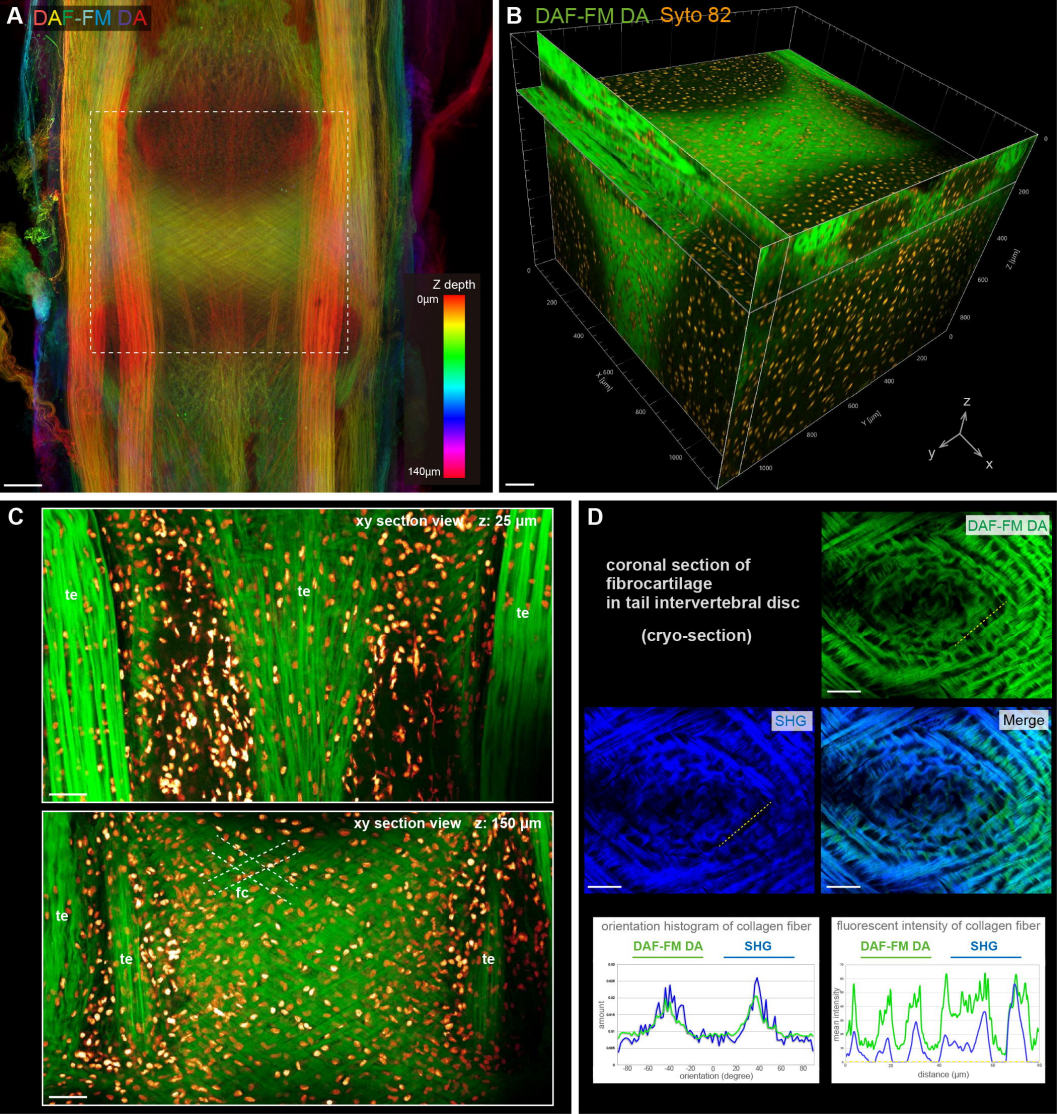
Fluorescent visualization of the collagen fibers in tendon and cartilage of postnatal mouse using DAF-FM DA. (**A**) The confocal fluorescent image with depth color-coded MIP of the tendon and cartilage in postnatal mouse tail stained with DAF-FM DA. (**B**) The 3D reconstructed confocal image in the area within the white dotted box of (A). The collagen fibers were stained with DAF-FM DA (green), and all nuclei were stained with Syto 82 (orange). (**C**) XY sectional views at an intervertebral region. te, tendon; fc, fibrocartilage. White dotted lines indicate the fibrocartilage orientation. (**D**) The fluorescent images of cryo-section samples at the tail intervertebral disc region. Orientation and fluorescent intensity of the collagen fibers visualized with DAF-FM DA (green) and SHG (blue) were plotted, respectively. Scale bar = 100 μm (A to C) and 40 μm (D).

We next investigated whether DAF-FM DA is useful in fluorescent visualization of collagen fibers in postnatal mice. To simultaneously observe cartilage and other collagen-rich tissues such as tendons, the tails of new-born mice were stained with DAF-FM DA. After incubation in DAF-FM DA solution, tail samples were cleared with transparency reagents. As a result, strong DAF-FM DA signals were observed in the tendon and in fibrocartilage of the intervertebral disc deep inside the tail (Fig. 4A). To simultaneously observe the distribution of collagen fibers and surrounding cells, nuclear staining was performed in the tail samples labelled with DAF-FM DA (Fig. 4B). Each XY cross-sectional image scanned by confocal microscopy displayed clearly labeled fiber structures in the tendon and intervertebral fibrocartilage (Fig. 4C). We observed that the nuclei were positioned in the gaps between fibers (Fig. 4C). We further confirmed that the fluorescence of DAF-FM DA overlapped well with the SHG signal in tissue sections of intervertebral discs (Fig. 4D). The orientation pattern of the fibers labelled with each fluorescence are approximately consistent (Fig. 4D). The signal intensity of SHG was not uniform and differed for each fiber, while the DAF-FM DA signal was detected uniformly throughout the fiber (Fig. 4D). In conclusion, we demonstrated that DAF-FM DA can be used to observe collagen fibers in postnatal mice tissues with high resolution enough to identify individual fibers by an easy method.

### Fluorescent visualization of collagen fibers in aquatic vertebrates using DAF-FM DA

To further demonstrate the utility of collagen fiber imaging using DAF-FM DA, we attempted DAF-FM DA staining in tissues of other vertebrate animals. Recently, Kuroda et al reported that DAF-FM DA fluorescently labels actinotrichia oriented in the tip of zebrafish fins (32). More recently, Ohashi et al reported that this probe fluorescently labels the collagen fiber network that develops three-dimensionally in the dermis of the axolotl (34). We tested whether this probe labels collagen fibers in other collagen-rich tissues by staining the whole bodies of these animals. In this experiment, living juvenile animals of zebrafish and axolotl were incubated in DAF-FM DA solution (fig. S6A and B). Firstly, we confirmed that DAF-FM DA fluorescently visualized the lattice-like collagen fiber structures that develop in the dermis of juvenile zebrafish and axolotl, similar to the recent report (fig. S6C and D). Additionally, we found that tendons at the myoseptum junction were visualized with strong fluorescence (fig. S6C). And also, clear fluorescence was observed in the tendons that develop in the joint regions of each fin bone in zebrafish (fig. S6C). Furthermore, tendons, as well as ligaments, in the digits of axolotl were visualized by DAF-FM DA with distinct signals (fig. S6F). These results demonstrate that DAF-FM DA is applicable for whole-body staining of collagen fibers in the tissues of living aquatic vertebrates.

### Pulse-chase observation using two-color fluorescent probes to analyze the growth dynamics of collagen fiber *in vitro* and *in vivo*

**Fig. 5.**
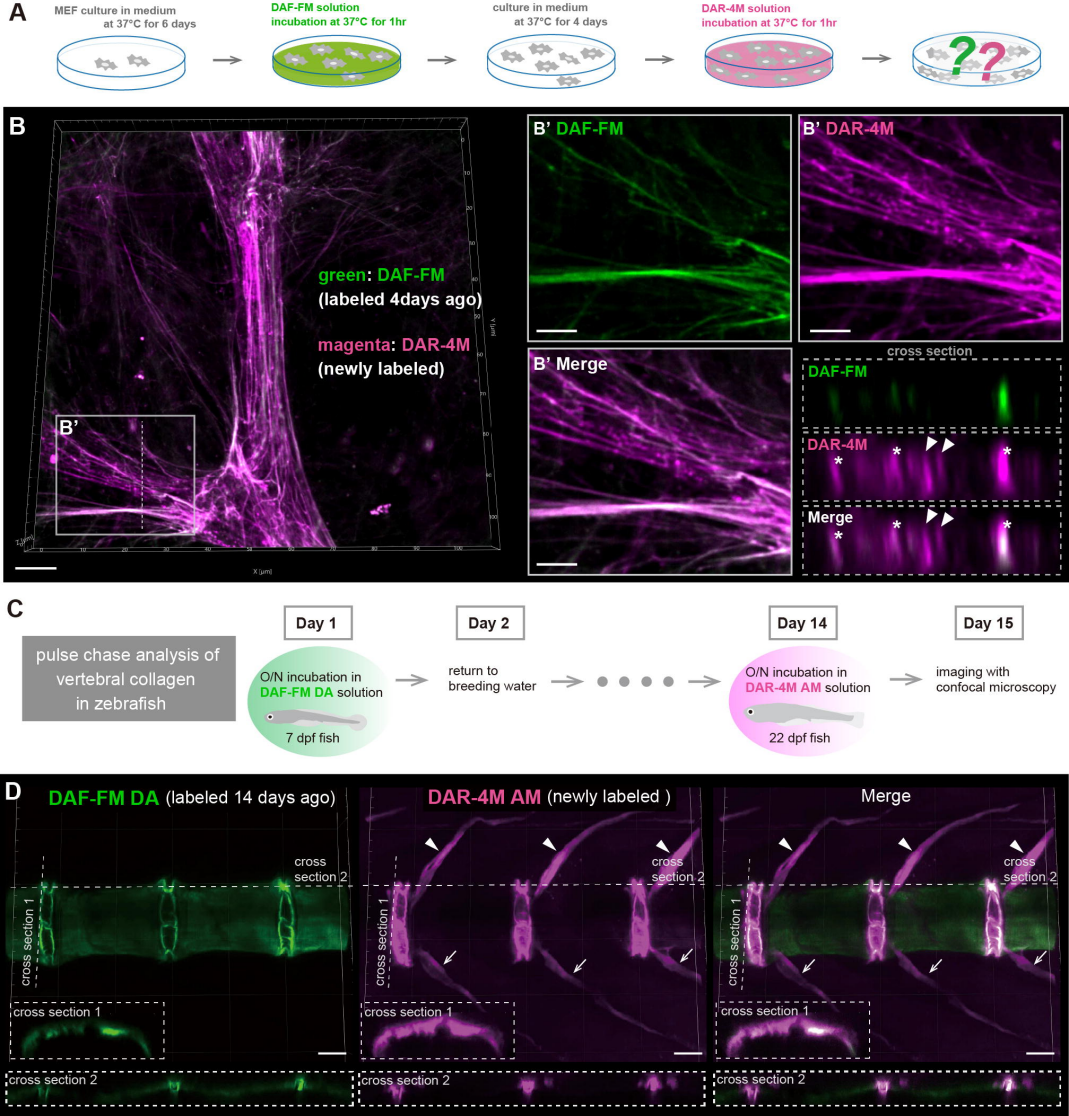
Pulse-chase observation using two different fluorescent probes to understand growth manner of collagen fibers *in vitro* and *in vivo*. (**A**) Schematic diagram illustrating the pulse-chase observation of the collagen fibers formed by MEFs, using DAF-FM and DAR-4M. MEF cultured for 6 days were first stained with DAF-FM. After DAF-FM staining, the staining solution was replaced with fresh medium and MEFs were incubated for 4 days. Subsequently, DAR-4M staining was performed, and the fluorescence of collagen fibers was observed. (**B**) Representative fluorescent images of the collagen fibers labeled by DAF-FM (green, labeled 4 days ago) and DAR-4M (magenta, newly labeled). Magnified images in the white box area and cross section images at the position of the white dotted line are shown in the right panels. Asterisks indicate the fibers that have thickened due to the additional growth of older fibers, and arrowheads indicate newly formed fibers between old fibers. (**C**) Schematic diagram illustrating the pulse-chase observation of the collagen fibers during the formation of vertebral bones in zebrafish, using DAF-FM DA and DAR-4M AM. Living zebrafish larvae at 7 dpf were first stained with DAF-FM DA. After the staining, the fish were returned to fresh tank water and bred for two weeks. Subsequently, DAR-4M AM staining was performed, and the fluorescence of the collagen fibers around the vertebrae was observed. (**D**) Representative fluorescent images of the collagen fibers around the vertebrae labeled by DAF-FM DA (green, labeled 14 days ago) and DAR-4M AM (magenta, newly labeled). Cross section images at the position of the white dotted lines in each fluorescent image are shown in the lower panels. Asterisks indicate the intervertebral regions. Arrowheads and arrows indicate neural spines and hemal spines, respectively. Scale bar = 10 μm (B), 5 μm (B’) and 50 μm (D).

We finally tested pulse-chase observation, which tracks the growth process of collagen fibers, using two fluorescent probes with different colors, DAF-FM and DAR-4M. For this experiment, it is essential that the fluorescence of the labeled fibers remains stable over an extended period without fading. Therefore, we fluorescently labeled collagen fibers with DAF-FM only once during MEF culture and then examined whether the fluorescence faded by temporal observations in the same area (fig. S7A). As a result, the fluorescence of collagen fibers labeled with DAF-FM hardly faded during the culture process after staining (fig. S7B). We then attempted the experiment to color-code collagen fibers during their growth by using a combination of the two fluorescent probes at different times during the culture process (Fig. 5A). As a result, we successfully distinguished between old fibers, formed four days ago, and newly formed fibers by performing DAF-FM and DAR-4M staining at different time points (Fig. 5B). Our result demonstrated the growth of a network of collagen fibers through the process of thickening by the deposition of new collagen around old fibers (Fig. 5B’ asterisk) and the formation of new fibers near old fibers (Fig. 5B’ arrowheads).

We further attempted to perform the pulse-chase observation of collagen fibers in living animal tissues. Here, we focused on the development of vertebrae in zebrafish. We first evaluated the reactivity of the two probes to the collagen fibers distributed in the notochord at the 7 days post fertilization (dpf) stage, before the start of vertebral calcification (fig. S8A). At the 7 dpf stage, strong fluorescence was detected in the notochord after DAF-FM DA staining, but only weak fluorescence was detected after DAR-4M AM (acetoxymethyl ester) staining (fig. S8A). On the other hand, at the stage of advanced calcification around the notochord associated with vertebral bone formation, strong fluorescence was detected in the intervertebral regions and in the neural and hemal spines after staining with both probes (fig. S8B). However, at this stage, no clear fluorescence was observed in the centrum regions with either probe (fig. S8B). We found that the fluorescence signals in the intervertebral regions and spines emitted with DAF-FM DA were similarly detected with collagen hybridizing peptide (CHP), a peptide that binds to the denatured collagen chains (44), staining after denaturation by heat treatment (fig. S8C). To clarify the dynamics in the distribution and production of collagen fibers during early osteogenesis in the vertebral region, we first incubated zebrafish larvae at the 7 dpf stage in DAF-FM DA solution and labeled the collagen fibers distributed at the notochord with green fluorescence (Fig. 5C). After staining with DAF-FM DA, the fish were bred in a circulating system tank for two weeks. Subsequently, the fish were incubated in DAR-4M AM solution (Fig. 5C). As a result, collagen fibers distributed at the notochord, initially labeled with DAF-FM DA, exhibited a remarkable change in their distribution pattern during 2 weeks of growth (Fig. 5D). The fluorescence of collagen fibers uniformly labeled at the notochord before bone formation emitted a strong signal specifically in the intervertebral disc region during osteogenesis (Fig. 5D). This fact indicates that collagen fibers uniformly distributed around the notochord at larval stage are reorganized during bone formation and reused as structurally specific components of the intervertebral disc. On the other hand, collagen fibers labeled with DAR-4M AM showed more extensive fluorescence in the intervertebral region compared to those with DAF-FM DA (Fig. 5D). Furthermore, no obvious signal was observed in the centrum region, whereas strong fluorescence labeled with DAR-4M AM was observed in the neural and hemal spines (Fig. 5D). This result suggests that in the early stages of vertebra formation, collagen production is more active in the spines and intervertebral regions than in the vertebral bodies. In summary, we successfully tracked the changes of collagen distribution pattern and visualized the active areas of collagen production in living zebrafish by applying the two probes at different times during the osteogenesis process. Hence, we demonstrated that DAF-FM DA and DAR-4M AM are useful for pulse-chase observation of collagen fibers during the growth of living tissues.

## DISCUSSION

In general, collagen fibers are distributed deep within animal tissues and form a three-dimensional network, making it difficult to stain them using whole mount tissues. Furthermore, due to the complexity of collagen structures, it is difficult to directly label collagen with fluorescent molecules, and many issues remain for imaging of collagen fibers using current technology. To overcome these issues, here we used cultured cells and vertebrate tissues to establish a method for visualizing collagen fibers with small-molecule fluorescent probes. One of the advantages of our imaging method is that it requires no special treatment prior to staining and the protocol is very simple. Even very thick tissues, such as the fibrocartilage in the intervertebral disc (Fig4), which is more than 300 μm thick, can be fluorescently stained clearly enough to distinguish the shape of individual fibers by simply immersing them overnight in a 10 μM DAF-FM DA dilute solution. In our method, observation of collagen fibers distributed deep within thick tissues such as cartilage and tendons in mice requires immersing the samples in a clearing reagent overnight after staining. However, DAF-FM emits via covalent bonding with allysine residues, so the fluorescence does not fade after transparency, making it a powerful imaging tool for three-dimensional visualization of fiber networks inside thick tissues. In addition, our technology has several other powerful advantages for the analysis of collagen dynamics. First, treatment with DAF-FM solutions at low concentrations of 5–10 μM does not exhibit cytotoxicity. Second, DAF-FM does not stain purified collagen or old collagen fibers that lack aldehyde groups. Instead, it binds to aldehyde groups in allysine residues before cross-linking occurs after secretion, enabling the staining of various fibrous collagens. Because DAF-FM covalently binds to the precursor of collagen cross-linking, the fluorescence of collagen fibers labeled with DAF-FM remains stable and hardly fade or diffuse after washout. And third, by utilizing the above-mentioned reaction specificity and applying two fluorescent probes with different colors at separate time points, it is possible to distinguish between old and newly formed collagen fibers that develop during their growth. Although several collagens labeled with fluorescent proteins have been developed for visualization (16–19, 45, 46), it is questionable whether such modified collagen with relatively large globular domains can fold and form fibrils normally. Additionally, this technique allows us to observe the distribution and morphology of collagen fibers only within a specific time frame and does not provide information on how individual fibers grow. On the other hand, our fluorescent tag specifically labels normally secreted collagen, and the tag is small enough compared to the fluorescent proteins to minimize interference with the fibril formation process. Combining DAF-FM and DAR-4M for pulse-chase observation is currently the only technique to overcome this problem, and we expect that it will be a breakthrough in future collagen research.

We revealed that DAF-FM and DAR-4M, known as detection probes for NO, label collagen fibers fluorescently in a manner independent of NO (fig. S3). Collagen fibers undergo a characteristic growth process in which three α-chains combine to form a single unit and these trimers polymerize through cross-link formation (5, 40). We successfully identified that DAF-FM covalently reacts to the aldehyde group of allysine residue, precursors of collagen cross-linking, located in the telopeptide domain of type I collagen by LC-MS analysis (Fig. 2E–G). Several recent studies focusing on LOX-mediated cross-link formation have proposed novel methods for detecting collagen fibers (21–25). In these methods, probes are immobilized by hydrazone or oxime formation targeting aldehyde groups of allysine residues for specific detection of collagen. While these chemical bond formations are in principle reversible reactions, DAF-FM reacts with aldehyde groups through an annulation reaction (Fig. 2H), which is thought to prevent elimination reactions effectively and result in long lifetime of labeling. In fact, fluorescence from DAF-FM-labelled collagen hardly faded for several days *in vitro* (fig. S7) and remained detectable for at least several days to weeks both *in vitro* and *in vivo* (Fig. 5). Furthermore, the fluorescence of DAF-FM-labelled collagen could be observed even after SDS-PAGE (fig. S5C) and transfer to membranes (Fig. 2D, fig. S5B). Other advantages of using DAF-FM and DAR-4M include that the probes are non-peptidic, low-molecular-weight compounds with high tissue permeability and that the two amine substituents on their benzene ring react with the targets to emit fluorescence, making them easy to use and minimizing interference from unreacted probes during fluorescence observation.

A very recent report has also shown that overnight treatment with low concentrations of DAF-FM DA does not adversely affect the growth of zebrafish tissues (32). However, if the aldehyde groups of lysine residues in the telopeptides of collagen molecules are masked by DAF-FM and its derivatives by more strong treatment, such as longer-term incubation with higher concentrations of the probes, the cross-link formation of collagen fibers might be inhibited. In order to perform *in vivo* pulse-chase analysis of the collagen fibers in various animal tissues in the future, it is necessary to verify the suitable experimental conditions that do not inhibit cross-link formation.

Several types of collagen molecules, including types I, II, III, V and XI, are known to form collagen fibers (4, 5, 47). All of these fibers polymerize and mature through a LOX-mediated cross-linking process (4, 5). In principle, DAF-FM and DAR-4M could label all these types of collagen fibers, and it is not possible to distinguish which type of collagen fiber is labeled with these probes. For the observation of tissues that simultaneously contain these types of collagen fibers, it may be necessary to use specific antibodies or other markers in combination to clarify which type of collagen fiber is stained. We found in this study that the reactivity of DAF-FM DA and DAR-4M AM, for the visualization of collagen fibers, varied depending on the developmental stage of animals (fig. S8). We speculate that DAR-4M AM also fluorescently labels collagen fibers via the same mechanism of action as DAF-FM DA, but the cause for the low reactivity of DAR-4M AM at some developmental stages is currently unknown. In addition, we evaluated the reactivity of the probes only in a limited number of animal tissues in the current study, and future validation of the reactivity of DAR-4M, in particular, is needed using tissues from various animal species. We also found that DAF-FM DA and DAR-4M AM do not uniformly label all collagen fibers in some tissues (Fig. 5, figS8). Considering the mechanism of action of these probes, it is possible that the collagen fibers at specific regions of the tissues with high LOX activity or rapid turnover could preferentially react with the probes and emit strong fluorescence, resulting in variations in labeling by the probes. In other words, this property can be utilized in the future as a tool for measuring LOX activity or collagen turnover rate in some tissues.

Previous techniques have been developed as a strategy to target collagen molecules themselves for the visualization of collagen fibers, but our method targets the intermediate structure of cross-linking, enabling clear fluorescent labeling of collagen fibers three-dimensionally oriented in animal tissues. In this study, we demonstrated that imaging with DAF-FM/DAR-4M is a powerful tool for the pulse-chase observation of collagen fibers in *in vitro* system using mammalian cells and in *in vivo* system using zebrafish. In the future, we expect to apply our method to the analysis of collagen fiber dynamics during mammalian tissue growth. Furthermore, our method could contribute to understanding the mechanisms of severe diseases such as fibrosis, which is caused by disruptions of the regulation of collagen fiber production and remodeling.

## MATERIALS AND METHODS

### Animal maintenance and tissue preparation

Preparation of mouse tissues was carried out at Osaka Medical and Pharmaceutical University. All mouse experiments were approved by the Institutional Review Board of Osaka Medical and Pharmaceutical University and performed in accordance with the Guide for the Animal Care and Use of Laboratory Animals of Osaka Medical and Pharmaceutical University. ICR mice were purchased from Japan SLC, Inc. (Shizuoka, Japan). Mice were euthanized under deep anesthesia using isoflurane inhalation for adults and hypothermia for pups and embryos. Tissues were harvested in phosphate buffered saline (PBS) and utilized for whole-mount staining or fixed with 4% paraformaldehyde in 0.1 M phosphate buffer (pH 7.4) for 2 days at 4°C. Zebrafish were maintained under the standard laboratory conditions and treated as previously described (48). AB strains were used as wild type zebrafish. Albino axolotls were raised and maintained under the conditions with 14 h of light/10L h of dark cycles at 20L °C and fed commercial granular solid food 2 times a day. Zebrafish and axolotls were anesthetized with tricaine (MS-222) at optimal concentrations according to each body size. All experiments using these animals were approved by the animal care and use at Osaka University.

### Microscopy and image analysis

The fluorescent images were obtained using confocal microscopes: LSM 780 (Carl Zeiss), STELLARIS8 (Leica), and FV1000 (Olympus), as well as a two-photon microscope, A1R MP+/Ti2-E (Nikon). 890 nm laser excitation and a 440 nm SP emission filter was used for SHG imaging of collagen fibers. ZEN (Carl Zeiss), LAS X (Leica), FV10-ASW (Olympus), and Fiji were used as image software for z projections. 3D image analysis was processed using Imaris 10.1.1 (Oxford Instruments). The fluorescence intensity values and orientation angles of collagen fibers were measured using FIJI.

### DAF-FM/DAR-4M staining for the visualization of collagen fibers formed by cultured cells

DAF-FM (Goryo Chemical, SK1003–01) and DAR-4M (Goryo Chemical, SK1005–01) were used for staining of the collagen fibers produced by mouse embryonic fibroblast (MEF) (Reprocell, RCHEFC003). The cells were cultured in Dulbecco’s Modified Eagle’s Medium (DMEM, FUJIFILM Wako Pure Chemical Corporation) supplemented with 10% fetal bovine serum (FBS), 200 μM L-ascorbic acid phosphate magnesium salt n-hydrate (FUJIFILM Wako Pure Chemical Corporation), 100 U/mL penicillin, and 100 μg/mL streptomycin (Sigma-Aldrich) at 37°C in a 5% CO_2_ atmosphere. 35 mm glass bottom dishes coated with 0.1% gelatin solution (Nacalai Tesque, 19895–75) were used for the MEF culture experiment. For the staining of collagen fibers deposited around the cultured cells, DAF-FM and DAR-4M were diluted with culture medium (DMEM with 10 % FBS) and adjusted to concentrations of 6.9 μM and 9.8 μM, respectively. Before staining with the DAF-FM or DAR-4M solutions, the cells were washed three times with DMEM without L-ascorbic acid phosphate magnesium salt n-hydrate. The cells were then incubated with the diluted DAF-FM or DAR-4M solutions for 1 hour at 37°C. After incubation, the samples stained with the probes were washed with fresh medium and observed using a confocal microscope. For the pulse-chase observation of collagen fibers formed *in vitro* culture condition, the cells were first incubated with DAF-FM solution, then after 4 days of culture with fresh medium, they were next incubated with DAR-4M solution. For the analysis using a confocal microscope, the fluorescent signals of the collagen fibers stained with DAF-FM and DAR-4M were detected with 488 nm and 561 nm lasers, respectively.

### DAF-FM staining of the purified collagen

DAF-FM (Goryo Chemical, SK1003–01) was used for staining of the purified collagen substrates. Gelatin (Nacalai Tesque, 19895–75), collagen type I (Nitta Gelatin, Cellmatrix Type I-P) and collagen type L (AteloCell, CL-22) were prepared on 35 mm glass bottom dishes as substrates for DAF-FM staining. Gelatin and collagen type L were adjusted to concentrations of 0.1% and 0.03 mg/ml, respectively, and coated on glass. Collagen type I was adjusted to a concentration of 1.8 mg/ml and neutralized by 1N NaOH to gel on glass. Each substrate was incubated with the DAF-FM solution diluted with DMEM with 10 % FBS, adjusted to a concentration of 6.9 μM, for 1 hour at 37°C. After the incubation, the samples stained with DAF-FM were washed with fresh medium and observed using a confocal microscope.

### DAF-FM DA/DAR-4M AM staining for the visualization of collagen fibers in vertebrate tissues

DAF-FM DA (Goryo Chemical, SK1004–01) and DAR-4M AM (Goryo Chemical, SK1006–01) were used for whole-mount staining to visualize vertebrate animal tissues. For the staining of whole-mount mouse tissues, DAF-FM DA was diluted with PBS, adjusted to concentrations of 5 μM, and used for staining. Mouse embryos or dissected tissues, including P0 - P1 skins and P14 tails, were incubated in the staining solution under dark conditions and treated for 12 hours at room temperature. After the staining, they were washed with PBS and fixed with 4% paraformaldehyde (PFA) in PBS O/N at 4°C. After the fixation, they were treated with RapiClear 1.52 (SJL, RC152001) O/N at room temperature for optical tissue clearing and observed using a confocal or two-photon microscope. For the staining of whole-mount juvenile of axolotls, DAF-FM DA and DAR-4M AM were diluted with breeding water, adjusted to concentrations of 5 μM and 10 μM, respectively, and used for staining. Living juvenile of axolotls were bathed in the staining solution under dark conditions and treated for 12 hours at 20L °C. After the staining, they were anesthetized with tricaine (MS-222) at an optimal concentration and fixed with 4% PFA in PBS O/N at 4°C. After the fixation, their skins were dissected and observed using a confocal microscope. Axolotl forelimbs were observed using a two-photon microscope after subsequent transparency treatment with RapiClear 1.49 (SJL, RC149001). For the staining of whole-mount zebrafish larvae, DAF-FM DA and DAR-4M AM were diluted with breeding water, adjusted to concentrations of 5 μM and 10 μM, respectively, and used for staining. Living zebrafish larvae were bathed in DAF-FM DA solution under dark conditions and treated for 12 hours at room temperature. After the staining, they were anesthetized with MS-222 at an optimal concentration and fixed with 4% PFA in PBS O/N at 4°C. After the fixation, their tissues including skins, tendons, and bones were observed with a confocal microscope. For the pulse-chase observation of vertebral collagen fibers, zebrafish larvae at 7dpf were first stained with DAF-FM DA, then after 2 weeks of breeding in a circulating tank, they were next stained with DAR-4M AM. For the observation using a confocal microscope, the fluorescent signals of the collagen fibers stained with DAF-FM DA and DAR-4M AM were detected with 488 nm and 561 nm lasers, respectively.

### Detection of DAF-FM modification of lysine at the telopeptide domains of type I collagen

The cell/matrix layers were sequentially digested with bacterial collagenase and pepsin to analyze DAF-FM modification of telopeptidyl lysine in type I collagen as previously reported (49). In brief, the samples were heated at 80°C for 30 minutes, and digestion with 0.01 mg/mL of recombinant collagenase from *Grimontia hollisae* (Nippi, Tokyo, Japan) (50) was performed in 100 mM Tris-HCl/5 mM CaCl_2_ (pH 7.5) at 37°C for 16 hours. After addition of acetic acid (final 0.5 M), digestion with 0.01 mg/mL of pepsin was further performed at 37°C for 16 hours. The peptide solutions were subjected to LC-MS analysis on a maXis II quadrupole time-of-flight mass spectrometer (Bruker Daltonics, Bremen, Germany) coupled to a Shimadzu Prominence UFLC-XR system (Shimadzu, Kyoto, Japan) using an Ascentis Express C18 HPLC column (5 μm particle size, L × I.D. 150 mm × 2.1 mm; Supelco, Bellefonte, PA, USA) (49). Peaks of peptides containing lysine or allysine labeled with DAF-FM (+392.061 Da) were detected in extracted ion chromatograms.

### Antibody staining of collagen

For the antibody staining of collagen type I, the cultured MEFs were fixed with 4% PFA in PBS and blocked with 1% bovine serum albumin (BSA)/PBS. After blocking, they were incubated with anti-mouse collagen type I rabbit polyclonal antibody (Rockland Inc, 600–401-103–0.1, 1:100 dilution) solution in 1% BSA/PBS O/N at 4°C. Next day, they were washed with PBS, and incubated with goat anti-rabbit IgG (H+L) antibody, FITC conjugate (Invitrogen, 65–6111, 1:200 dilution) solution in 1% BSA/PBS for 2 hours at room temperature. For the antibody staining of collagen type , the mouse E14.5 embryos were fixed with 4% PFA in PBS. After fixation, their tails were dissected and sequentially immersed in 10%, 20%, and 30% sucrose solutions in PBS and in a 1:1 solution of 30% sucrose and Tissue-Tek O.C.T Compound (Sakura Finetek Japan, 4583). Subsequently, they were embedded in O.C.T Compound and frozen using dry ice. The tissues were then sectioned at a thickness of 10 μm using a cryomicrotome CM1850 (Leica). The cryo-section samples were blocked with 2% bovine serum albumin (BSA)/PBS. After blocking, they were incubated with anti-chick collagen type L mouse monoclonal antibody (DSHB, L-L6B3, 1:100 dilution) solution in 2% BSA/PBS O/N at 4°C. Next day, they were washed with PBS and incubated with goat anti-mouse IgG (H+L) antibody, Alexa 594 conjugate (Invitrogen, A11020, 1:200 dilution) solution in 1% BSA/PBS for 1 hour at room temperature. Immunofluorescence images were obtained using a confocal microscope.

### Staining with denatured collagen-binding peptide

For staining of the collagen fibers deposited around cultured cells, the cells were treated with phosphate-buffered saline (PBS) heated at 95°C for 1 minute to heat-denature extracellular collagen. They were then fixed with a 4% PFA and blocked with 2% BSA/PBS. After blocking, they were incubated with 5 μg/mL of Bin*d*COL, biotin-conjugated (Funakoshi, FDV-0035) in 1% BSA/PBS or 3 μg/mL of fluorescein-conjugated soCMP6–7(Glu)2 in 1% BSA/PBS O/N at 4°C and washed with PBS (35). The cell samples incubated with Bin*d*COL solution were stained with streptavidin, Alexa 647 conjugate (Invitrogen, S32357, 1:200 dilution) solution in 1% BSA/PBS for 1 hour at room temperature and washed with PBS after staining. Finally, the fluorescence signals of denatured collagen fibers were imaged using a confocal microscope. For staining of the collagen fibers distributed in zebrafish tissues, the tissues were fixed with a 4% PFA. After fixation, they were heat-treated in a thermo bath at 80°C for 10 minutes to denature extracellular collagen. They were then blocked with 2% BSA/PBS and incubated with 5 μM of CHP-Cy3 in 2% BSA/PBS O/N at 4°C, and then washed with PBS. Finally, the fluorescence signals of denatured collagen fibers were imaged using a confocal microscope.

### Staining of collagen deposited around BAPN-treated cultured cells

A previously established MEF clones were used in this staining (51). The cells were cultured in DMEM with 10% FBS and 200 μM L-ascorbic acid phosphate magnesium salt n-hydrate at 37°C in a 5% CO_2_ atmosphere. The medium was replaced with HFDM-1(+) (Cell Science & Technology Institute Inc.) containing 100 U/mL penicillin and 100 μg/mL streptomycin after the cells had reached confluence in 35 mm glass-bottom dishes. Confluent MEFs were incubated in HFDM-1(+) with or without the addition of 500 μM 3-aminopropionitrile fumarate (BAPN, Sigma-Aldrich) for 2 days in 35 mm glass-bottom dishes. Subsequently, the cells were washed with PBS and incubated in a medium containing DAF-FM/DMSO (final concentration of 6.9 μM DAF-FM, and 0.1% DMSO) or DMSO (final concentration of 0.1%) at a dilution of 1/1000 for an additional hour. In cases where BAPN was added, it was introduced to a concentration of 500 μM. After washing the cells with PBS, they were fixed in a 4% paraformaldehyde phosphate buffer solution for 10 minutes. The cells were washed with PBS and observed using a confocal laser microscope.

### Staining of cellular actin and nucleus

For staining the actin cytoskeleton and cell nuclei of the cultured MEFs, the cells were fixed with 4% PFA. After fixation, they were washed with PBS and incubated with a solution of Phalloidin-iFluor 594 conjugated (AAT Bioquest; 1:300 dilution) and Hoechst (Dojindo; 1:500 dilution) in PBS for 2 hours at room temperature. For staining of cell nuclei in frozen sections of mouse tail tissues, the samples were fixed with 4% PFA. After fixation, they were washed with PBS and incubated with a solution of Hoechst (Dojindo; 1:500 dilution) in PBS O/N at 4°C. For staining of cell nuclei in P14 mouse tail and P1 mouse skin, the tissue samples were fixed with 4% PFA. After fixation, they were washed with PBS and incubated with a solution of Syto 82 (Invitrogen; 1:1000 dilution) in PBS O/N at 4°C. Each sample was washed in PBS after staining, and nuclear fluorescence was captured using confocal microscopy.

### BrdU incorporation assay

MEFs were incubated in culture medium for 6 days and then incubated in DAF-FM solution at a concentration of 6.9 μM for 1 hour. After staining with DAF-FM, cells were washed with fresh medium and treated for 12 hours in medium containing BrdU (Abcam, ab142567) adjusted to a concentration of 10 μM. Cells were fixed with cold 70% EtOH for 5 minutes at room temperature, and then treated with 0.1N HCl solution containing 0.1% Triton for 30 minutes at 37°C to increase permeability of the cell nuclei. After several washes in PBS, cell samples were blocked for 1 hour with a 1% BSA/PBS solution for antibody staining. Mouse monoclonal anti-BrdU antibody (Molecular Probe, A21300, 1:200 dilution) was used as the primary antibody, and cell samples were incubated O/N at 4°C in the antibody solution containing 1% BSA/PBS. The next day, they were washed with PBS and incubated with goat anti-mouse IgG antibody, Alexa 594 conjugated (Invitrogen, A11020, 1:200 dilution) solution with 1% BSA/PBS for 2 hours at room temperature. Cell nuclei were simultaneously stained with Hoechst (Dojindo; 1:500 dilution) and the percentage of BrdU-positive nuclei was compared between cells without DAF-FM staining (control) and cells with DAF-FM staining.

### Cell viability assay

MEFs were incubated in culture medium for 7 days and then incubated with DAF-FM solution at a concentration of 6.9 μM for 1 hour. After staining with DAF-FM, cells were washed in fresh medium and incubated in Syto 82 (Invitrogen, S11363, 1:1000 dilution) solution at 37°C for 30 minutes to label the nuclei of living cells. Cells were then washed in fresh medium and incubated in Nuclear Blue (AAT Bioquest, Live or Dead Cell Viability Assay Kit, 22788, 1:200 dilution) solution for 30 minutes at 37°C to label the nuclei of dead cells. After nuclear staining, cells were washed with PBS, and the percentage of Nuclear Blue-positive nuclei was compared between cells without DAF-FM staining (control) and cells with DAF-FM staining.

### Drug treatment for NO removal and NOS inhibition

MEFs were cultured for 10 days in the culture medium as described above. They were then treated under the following three different conditions: DMSO (0.1% in DMEM) as control, 2-(4-Carboxyphenyl)-4,4,5,5-tetramethylimidazoline-1-oxyl-3-oxide (C-PTIO, Dojindo) (500 μM in DMEM) known as a NO remover (52), and N^G^-nitro-L-arginine methyl ester hydrochloride (L-NAME, Dojindo) (500 μM in DMEM) known as an inhibitor of NOS (53). In the control and L-NAME treatment experiment, after 24 hours of treatment, MEFs were incubated for 1 hour at 37°C in DAF-FM solution (6.9 μM) diluted with DMEM containing the corresponding drug (0.1% DMSO and 500 μM L-NAME). In C-PTIO treatment experiment, after 30 minutes of the treatment, MEFs were incubated in DAF-FM solution (6.9 μM) diluted with DMEM containing C-PTIO (500 μM) for 1 hour at 37°C. After DAF-FM staining, fluorescent images of the collagen fibers produced by MEFs were imaged using a confocal microscope and the fluorescence intensity values were measured by FIJI image analysis software.

### Western blot analysis of collagen in cell layers

As in the experiment of staining collagen deposited around BAPN-treated cultured cells, confluent MEFs in 35 mm dishes were incubated in HFDM-1(+) with or without BAPN for 2 days and then treated with DAF-FM/DMSO or DMSO for 1 hour. Following a wash with PBS, the cell layers were dissolved in SDS-PAGE sample buffer (50 mM Tris-HCl [pH 6.7], 10% glycerol, and 2% SDS) and heated at 95°C for 5 minutes. The protein concentration of these SDS samples was determined using Pierce^™^ BCA protein assay kit (Thermo Fisher Scientific, Waltham, MA, USA). SDS-PAGE was conducted on a 5% polyacrylamide gel with 91 mM 1,4-dithiothreitol (DTT)-reduced or non-reduced samples, and proteins on the gel were transferred to nitrocellulose membranes. Fluorescent bands were visualized with a CCD imager LAS-3000 (Fujifilm, Tokyo, Japan). Subsequently, the membranes were blocked with 5% skim milk/Tris-buffered saline (TBS; 50 mM Tris-HCl pH 7.4, 150 mM NaCl) and washed with TBS. They were treated with 1 μg/mL of biotin-conjugated Bin*d*COL in 2% skim milk/TBS to detect collagen polypeptides (35). The membranes were washed with TBS, treated with streptavidin-HRP (Thermo Fisher Scientific, 1:5000 dilution) in 2% skim milk/TBS, and washed with TBS containing 0.1% Tween-20. Collagen bands were detected with a CCD imager LAS-3000 using Pierce^™^ ECL western blotting substrate kit (Thermo Fisher Scientific).

### SDS-PAGE analysis of cell layers treated with pepsin

Confluent MEFs in 35 mm dishes were incubated in HFDM-1(+) containing DAF-FM/DMSO (final concentration 6.9 μM DAF-FM, 0.1% DMSO) or DMSO (final concentration 0.1% DMSO) for 2 days. The cell layers were washed with PBS and those collected with cell scrapers were treated with 0.1 M HCl, with or without 100 μg/mL pepsin (Sigma-Aldrich), at 4°C for 16 hours. After neutralization with NaOH, these samples were mixed with 5 × SDS sample buffer and heated at 95°C for 5 minutes. Proteins in 91 mM DTT-reduced or non-reduced samples were separated by SDS-PAGE on an 8% polyacrylamide gel, and fluorescent bands were visualized with a CCD imager LAS-3000. Subsequently, protein bands were visualized by Coomassie Brilliant Blue R-250 staining.

### Statistical analysis

Statistical analyses were performed using GraphPad Prism version 9.5.1 (731). All data are presented as mean ± SD. An unpaired two-tailed Student’s t-test was used to assess the statistical significance of the differences between the means of two independent groups. ANOVA followed by Tukey’s multiple comparison test was conducted to evaluate the statistical significance of differences among the three groups.

## Supplementary Material

Supplement 1 **fig. S1. Reactivity of DAF-FM with purified collagen.** (**A**) Schematic diagram of DAF-FM staining for the purified collagen substrates. (**B**) Representative confocal images of the purified collagen after DAF-FM staining. Scale bar = 50 μm.

Supplement 2 **fig. S2. Fluorescent staining of the collagen fibers formed by culture cells using DAR-4M.** (**A**) Schematic diagram of DAR-4M staining for the collagen fibers formed by MEFs. (**B**) Upper panels show representative fluorescent images of the collagen fibers visualized with DAF-FM (green) and DAR-4M (magenta) at culture day 10. Both probes visualized the same fibers. Lower panels show representative fluorescent images of the collagen fibers visualized with DAR-4M (magenta) and anti-Col1 antibody staining at culture day 10. The fluorescent signals of DAR-4M and anti-Col1 antibody staining were merged well on the same fibers. Scale bar = 50 μm.

Supplement 3 **fig. S3. NO-independent fluorescent visualization of collagen fibers by DAF-FM.** (**A**) Schematic diagram of DAF-FM staining under NO removal conditions for the collagen fibers formed by MEFs. (**B**) Representative fluorescent images of the collagen fibers visualized with DAF-FM (green) at culture day 10. Clear fluorescent signals of collagen fibers stained with DAF-FM were detected under C-PTIO (NO removal) and L-NAME (NOS inhibition) treatment conditions, similar to the control. The fluorescence intensity plots for each condition are shown in the right panel. Scale bar = 50 μm.

Supplement 4 **fig. S4. No negative effects on cell activities with DAF-FM treatment.** (**A**) Experimental workflow to investigate the effect of DAF-FM treatment on cell division. (**B**) Representative images of anti-BrdU antibody staining. Cultured MEFs were incubated with DMSO (control) or DAF-FM solution. All cell nuclei were stained with Hoechst (blue), and cell nuclei incorporating BrdU were stained with anti-BrdU antibody (magenta). Collagen fibers were stained with DAF-FM (green). (**C**) Number of BrdU-positive cells was counted under each condition. There was no significant difference in the percentage of BrdU-positive cells between the conditions. (**D**) Experimental workflow to investigate the effect of DAF-FM treatment on cell death. (**E**) Representative images of Nuclear Blue staining. Cultured MEFs were incubated with DMSO (control) or DAF-FM solution. All cell nuclei were stained with Syto 82 (orange), and cell nuclei of apoptotic cells were stained with Nuclear Blue (blue). Collagen fibers were stained with DAF-FM (green). (**F**) Number of apoptotic cells was counted under each condition. There was no significant difference in the percentage of apoptotic cells between the conditions. Scale bar = 50 μm.

Supplement 5 **fig. S5. DAF-FM fluorescence suppressed by inhibition of collagen cross-linking formation** (**A**) Representative fluorescent images of the collagen fibers labeled by DAF-FM (green) under the various culture conditions. DAF-FM fluorescence of the collagen fibers produced by MEFs is not suppressed when BAPN is present only during DAF-FM staining, but it is significantly suppressed when BAPN is present during MEF culture. Scale bar = 50 μm. (**B**) SDS-PAGE analysis of DAF-FM-labeled collagen with or without DTT. DAF-FM fluorescence of proteins produced by MEFs in the presence or absence of BAPN was examined (upper panel), and Bin*d*COL staining of the same protein samples was performed (lower panel). (**C**) DAF-FM fluorescence of pepsin-digested or undigested proteins produced by MEFs was examined (upper panel), and CBB staining of the same protein samples was performed (lower panel).

Supplement 6 **fig. S6. Fluorescent visualization of collagen fibers in zebrafish and axolotl using DAF-FM DA.** (**A, B**) Schematic diagram of DAF-FM DA staining for the collagen fibers in zebrafish and axolotl. Living juvenile zebrafish and axolotl were incubated overnight in 10μM DAF-FM solution. After the incubation, DAF-FM DA fluorescence in the animal tissues were imaged by a confocal microscopy. (**C, D**) Representative fluorescent image with depth color-coded MIP of the body skin labeled by DAF-FM DA in zebrafish and axolotl, respectively. The magnified MIPs of the areas within the white boxes are shown in the right panels of each image. White dotted lines indicate the orientation of collagen fibers. (**E**) Representative fluorescent images of the tendon labeled by DAF-FM (green) in the zebrafish fin bones. Fin bones were stained with Alizarin Red (magenta). (**F**) Representative fluorescent images of the tendon and ligament labeled by DAF-FM DA (green) in the axolotl forelimb digits. te, tendon; li, ligament. Scale bar = 50 μm (B, C and F) and 200 μm (E).

Supplement 7 **fig. S7. Fluorescence of the collagen fibers labeled with DAF-FM hardly fade after washout.** (**A**) Schematic diagram of the temporal observation after DAF-FM staining for the collagen fibers formed by MEFs. (**B**) Representative fluorescent images of the collagen fibers labeled with DAF-FM at day 0, day 2 and day4 after labeling. Fluorescent mean intensity values of the DAF-FM at each time point are shown in the right panel. Scale bar = 100 μm.

Supplement 8 **fig. S8. Reactivity of DAF-FM DA and DAR-4M AM with zebrafish notochord and vertebral bones.** (**A**) Representative confocal images of the collagen fibers in the notochord at 7 dpf simultaneously labeled with DAF-FM DA and DAR-4M AM. Each image with fluorescence intensity color-coded MIPs is shown in the lower panels. (**B**) Representative confocal images of the collagen fibers in the intervertebral regions and spines at 20 dpf simultaneously labeled with DAF-FM DA and DAR-4M AM. Each image with fluorescence intensity color-coded MIPs is shown in the lower panels. (**C**) Representative confocal images of the collagen fibers in the intervertebral regions and spines at 24 dpf co-labeled with DAF-FM DA and CHP-Cy3. Cross section images at the position of the white dotted lines in each fluorescent image are shown in the lower panels. nt, notochord; ns, neural spine; hs, hemal spine. Open arrowheads indicate the myoseptum tendon, arrows indicate unknown nonspecific signals detected in DAR-4M AM labeled samples, and asterisks indicate the intervertebral discs. Scale bar = 50 μm.

**Supplemental materials:** This article contains supplemental materials.

## References

[1] Keeley F. W., Mecham R. P., Eds., Evolution of Extracellular Matrix (Springer Berlin Heidelberg, Berlin, Heidelberg, 2013; https://link.springer.com/10.1007/978-3-642-36002-2)Biology of Extracellular Matrix.

[2] Frantz C., Stewart K. M., Weaver V. M., The extracellular matrix at a glance. Journal of Cell Science 123, 4195–4200 (2010). 21123617 10.1242/jcs.023820 PMC2995612

[3] Kadler K. E., Baldock C., Bella J., Boot-Handford R. P., Collagens at a glance. Journal of Cell Science 120, 1955–1958 (2007). 17550969 10.1242/jcs.03453

[4] Ricard-Blum S., The Collagen Family. Cold Spring Harbor Perspectives in Biology 3, a004978–a004978 (2011). 21421911 10.1101/cshperspect.a004978 PMC3003457

[5] Mouw J. K., Ou G., Weaver V. M., Extracellular matrix assembly: a multiscale deconstruction. Nat Rev Mol Cell Biol 15, 771–785 (2014). 25370693 10.1038/nrm3902 PMC4682873

[6] Fidler A. L., Boudko S. P., Rokas A., Hudson B. G., The triple helix of collagens – an ancient protein structure that enabled animal multicellularity and tissue evolution. Journal of Cell Science 131, jcs203950 (2018). 29632050 10.1242/jcs.203950 PMC5963836

[7] Kuivaniemi H., Tromp G., Prockop D. J., Mutations in fibrillar collagens (types I, II, III, and XI), fibril-associated collagen (type IX), and network-forming collagen (type X) cause a spectrum of diseases of bone, cartilage, and blood vessels. Hum. Mutat. 9, 300–315 (1997). 9101290 10.1002/(SICI)1098-1004(1997)9:4<300::AID-HUMU2>3.0.CO;2-9

[8] Etich J., Leßmeier L., Rehberg M., Sill H., Zaucke F., Netzer C., Semler O., Osteogenesis imperfecta-pathophysiology and therapeutic options. Mol Cell Pediatr 7, 9 (2020). 32797291 10.1186/s40348-020-00101-9 PMC7427672

[9] Wynn T. A., Ramalingam T. R., Mechanisms of fibrosis: therapeutic translation for fibrotic disease. Nat Med 18, 1028–1040 (2012). 22772564 10.1038/nm.2807 PMC3405917

[10] Dooling L. J., Saini K., Anlaş A. A., Discher D. E., Tissue mechanics coevolves with fibrillar matrisomes in healthy and fibrotic tissues. Matrix Biol 111, 153–188 (2022). 35764212 10.1016/j.matbio.2022.06.006 PMC9990088

[11] Goodyear R. J., Lu X., Deans M. R., Richardson G. P., A tectorin-based matrix and planar-cell-polarity genes are required for normal collagen-fibril orientation in the developing tectorial membrane. Development, dev.151696 (2017). 10.1242/dev.151696 PMC5702074 28935705

[12] Seidel R., Blumer M., Pechriggl E.-J., Lyons K., Hall B. K., Fratzl P., Weaver J. C., Dean M. N., Calcified cartilage or bone? Collagens in the tessellated endoskeletons of cartilaginous fish (sharks and rays). Journal of Structural Biology 200, 54–71 (2017). 28923317 10.1016/j.jsb.2017.09.005

[13] Mayorca-Guiliani A. E., Willacy O., Madsen C. D., Rafaeva M., Elisabeth Heumüller S., Bock F., Sengle G., Koch M., Imhof T., Zaucke F., Wagener R., Sasaki T., Erler J. T., Reuten R., Decellularization and antibody staining of mouse tissues to map native extracellular matrix structures in 3D. Nat Protoc 14, 3395–3425 (2019). 31705125 10.1038/s41596-019-0225-8

[14] Campagnola P. J., Millard A. C., Terasaki M., Hoppe P. E., Malone C. J., Mohler W. A., Three-Dimensional High-Resolution Second-Harmonic Generation Imaging of Endogenous Structural Proteins in Biological Tissues. Biophysical Journal 82, 493–508 (2002). 11751336 10.1016/S0006-3495(02)75414-3 PMC1302489

[15] Chen X., Nadiarynkh O., Plotnikov S., Campagnola P. J., Second harmonic generation microscopy for quantitative analysis of collagen fibrillar structure. Nat Protoc 7, 654–669 (2012). 22402635 10.1038/nprot.2012.009 PMC4337962

[16] Morris J. L., Cross S. J., Lu Y., Kadler K. E., Lu Y., Dallas S. L., Martin P., Live imaging of collagen deposition during skin development and repair in a collagen I – GFP fusion transgenic zebrafish line. Developmental Biology 441, 4–11 (2018). 29883658 10.1016/j.ydbio.2018.06.001 PMC6080847

[17] Shiflett L. A., Tiede-Lewis L. M., Xie Y., Lu Y., Ray E. C., Dallas S. L., Collagen Dynamics During the Process of Osteocyte Embedding and Mineralization. Front. Cell Dev. Biol. 7, 178 (2019). 31620436 10.3389/fcell.2019.00178 PMC6759523

[18] Kashimoto R., Furukawa S., Yamamoto S., Kamei Y., Sakamoto J., Nonaka S., Watanabe T. M., Sakamoto T., Sakamoto H., Satoh A., Lattice-patterned collagen fibers and their dynamics in axolotl skin regeneration. iScience 25, 104524 (2022). 35754731 10.1016/j.isci.2022.104524 PMC9213773

[19] Hino H., Kondo S., Kuroda J., In vivo imaging of bone collagen dynamics in zebrafish. Bone Rep 20, 101748 (2024). 38525199 10.1016/j.bonr.2024.101748 PMC10959726

[20] Timin G., Milinkovitch M. C., High-resolution confocal and light-sheet imaging of collagen 3D network architecture in very large samples. iScience 26, 106452 (2023). 37020961 10.1016/j.isci.2023.106452 PMC10067766

[21] Aronoff M. R., Hiebert P., Hentzen N. B., Werner S., Wennemers H., Imaging and targeting LOX-mediated tissue remodeling with a reactive collagen peptide. Nat Chem Biol 17, 865–871 (2021). 34253910 10.1038/s41589-021-00830-6

[22] Wang H., Poe A., Pak L., Nandakumar K., Jandu S., Steppan J., Löser R., Santhanam L., An in situ activity assay for lysyl oxidases. Commun Biol 4, 840 (2021). 34226627 10.1038/s42003-021-02354-0 PMC8257687

[23] Ma H., Zhou I. Y., Chen Y. I., Rotile N. J., Ay I., Akam E. A., Wang H., Knipe R. S., Hariri L. P., Zhang C., Drummond M., Pantazopoulos P., Moon B. F., Boice A. T., Zygmont S. E., Weigand-Whittier J., Sojoodi M., Gonzalez-Villalobos R. A., Hansen M. K., Tanabe K. K., Caravan P., Tailored Chemical Reactivity Probes for Systemic Imaging of Aldehydes in Fibroproliferative Diseases. J Am Chem Soc 145, 20825–20836 (2023). 37589185 10.1021/jacs.3c04964 PMC11022681

[24] Hiebert P., Antoniazzi G., Aronoff M., Werner S., Wennemers H., A lysyl oxidase-responsive collagen peptide illuminates collagen remodeling in wound healing. Matrix Biol 128, 11–20 (2024). 38382767 10.1016/j.matbio.2024.02.006

[25] Akam-Baxter E. A., Bergemann D., Ridley S. J., To S., Andrea B., Moon B., Ma H., Zhou Y., Aguirre A., Caravan P., Gonzalez-Rosa J. M., Sosnovik D. E., Dynamics of collagen oxidation and cross linking in regenerating and irreversibly infarcted myocardium. Nat Commun 15, 4648 (2024). 38858347 10.1038/s41467-024-48604-7 PMC11164919

[26] Kojima H., Nakatsubo N., Kikuchi K., Kawahara S., Kirino Y., Nagoshi H., Hirata Y., Nagano T., Detection and Imaging of Nitric Oxide with Novel Fluorescent Indicators: Diaminofluoresceins. Anal. Chem. 70, 2446–2453 (1998). 9666719 10.1021/ac9801723

[27] Kojima H., Urano Y., Kikuchi K., Higuchi T., Hirata Y., Nagano T., Fluorescent Indicators for Imaging Nitric Oxide Production. Angew. Chem. Int. Ed. 38, 3209–3212 (1999). 10.1002/(sici)1521-3773(19991102)38:21<3209::aid-anie3209>3.0.co;2-6 10556905

[28] Kojima H., Hirotani M., Nakatsubo N., Kikuchi K., Urano Y., Higuchi T., Hirata Y., Nagano T., Bioimaging of Nitric Oxide with Fluorescent Indicators Based on the Rhodamine Chromophore. Anal. Chem. 73, 1967–1973 (2001). 11354477 10.1021/ac001136i

[29] Lepiller S., Laurens V., Bouchot A., Herbomel P., Solary E., Chluba J., Imaging of nitric oxide in a living vertebrate using a diaminofluorescein probe. Free Radical Biology and Medicine 43, 619–627 (2007). 17640572 10.1016/j.freeradbiomed.2007.05.025

[30] Renn J., Pruvot B., Muller M., Detection of nitric oxide by diaminofluorescein visualizes the skeleton in living zebrafish. J. Appl. Ichthyol. 30, 701–706 (2014).

[31] Huysseune A., Larsen U. G., Larionova D., Matthiesen C. L., Petersen S. V., Muller M., Witten P. E., Bone Formation in Zebrafish: The Significance of DAF-FM DA Staining for Nitric Oxide Detection. Biomolecules 13, 1780 (2023). 38136650 10.3390/biom13121780 PMC10742054

[32] Kuroda J., Hino H., Kondo S., Dynamics of actinotrichia, fibrous collagen structures in zebrafish fin tissues, unveiled by novel fluorescent probes. PNAS Nexus 3, pgae266 (2024). 39296332 10.1093/pnasnexus/pgae266 PMC11409509

[33] Miyamoto K., Kuroda J., Kamimura S., Sasano Y., Abe G., Ansai S., Funayama N., Uesaka M., Tamura K., Actinotrichia-independent developmental mechanisms of spiny rays facilitate the morphological diversification of Acanthomorpha fish fins. bioRxiv [Preprint] (2025). 10.1101/2025.03.01.640274. PMC13018208 41690953

[34] Ohashi A., Sakamoto H., Kuroda J., Kondo Y., Kamei Y., Nonaka S., Furukawa S., Yamamoto S., Satoh A., Keratinocyte-driven dermal collagen formation in the axolotl skin. Nat Commun 16, 1757 (2025). 39994199 10.1038/s41467-025-57055-7 PMC11850728

[35] Takita K. K., Fujii K. K., Ishii K., Koide T., Structural optimization of cyclic peptides that efficiently detect denatured collagen. Org Biomol Chem 17, 7380–7387 (2019). 31342036 10.1039/c9ob01042d

[36] Li H., Wan A., Fluorescent probes for real-time measurement of nitric oxide in living cells. Analyst 140, 7129–7141 (2015). 26373251 10.1039/c5an01628b

[37] Nasuno R., Yoshikawa Y., Takagi H., Acetaldehyde reacts with a fluorescent nitric oxide probe harboring an o-phenylenediamine structure that interferes with fluorometry. Free Radical Biology and Medicine 187, 29–37 (2022). 35605899 10.1016/j.freeradbiomed.2022.05.014

[38] Rucker R., Kosonen T., Clegg M., Mitchell A., Rucker B., Uriu-Hare J., Keen C., Copper, lysyl oxidase, and extracellular matrix protein cross-linking. The American Journal of Clinical Nutrition 67, 996S–1002S (1998). 9587142 10.1093/ajcn/67.5.996S

[39] Vallet S. D., Ricard-Blum S., Lysyl oxidases: from enzyme activity to extracellular matrix cross-links. Essays in Biochemistry 63, 349–364 (2019). 31488698 10.1042/EBC20180050

[40] Shoulders M. D., Raines R. T., Collagen structure and stability. Annu Rev Biochem 78, 929–958 (2009). 19344236 10.1146/annurev.biochem.77.032207.120833 PMC2846778

[41] Tang S. S., Trackman P. C., Kagan H. M., Reaction of aortic lysyl oxidase with beta-aminopropionitrile. J Biol Chem 258, 4331–4338 (1983). 6131892

[42] Terajima M., Taga Y., Cabral W. A., Liu Y., Nagasawa M., Sumida N., Kayashima Y., Chandrasekaran P., Han L., Maeda N., Perdivara I., Hattori S., Marini J. C., Yamauchi M., Cyclophilin B control of lysine post-translational modifications of skin type I collagen. PLOS Genetics 15, e1008196 (2019). 31173582 10.1371/journal.pgen.1008196 PMC6602281

[43] Saito T., Terajima M., Taga Y., Hayashi F., Oshima S., Kasamatsu A., Okubo Y., Ito C., Toshimori K., Sunohara M., Tanzawa H., Uzawa K., Yamauchi M., Decrease of lysyl hydroxylase 2 activity causes abnormal collagen molecular phenotypes, defective mineralization and compromised mechanical properties of bone. Bone 154, 116242 (2022). 34718219 10.1016/j.bone.2021.116242

[44] Hwang J., Huang Y., Burwell T. J., Peterson N. C., Connor J., Weiss S. J., Yu S. M., Li Y., In Situ Imaging of Tissue Remodeling with Collagen Hybridizing Peptides. ACS Nano 11, 9825–9835 (2017). 28877431 10.1021/acsnano.7b03150 PMC5656977

[45] Lu Y., Kamel-El Sayed S. A., Wang K., Tiede-Lewis L. M., Grillo M. A., Veno P. A., Dusevich V., Phillips C. L., Bonewald L. F., Dallas S. L., Live Imaging of Type I Collagen Assembly Dynamics in Osteoblasts Stably Expressing GFP and mCherry-Tagged Collagen Constructs. J of Bone & Mineral Res 33, 1166–1182 (2018). 10.1002/jbmr.3409 PMC6425932 29461659

[46] Tanaka T., Moriya K., Tsunenaga M., Yanagawa T., Morita H., Minowa T., Tagawa Y.-I., Hanagata N., Inagaki Y., Ikoma T., Visualized procollagen Iα1 demonstrates the intracellular processing of propeptides. Life Sci Alliance 5, e202101060 (2022). 35181633 10.26508/lsa.202101060 PMC8860094

[47] Van Der Rest M., Garrone R., Collagen family of proteins. The FASEB Journal 5, 2814–2823 (1991). 1916105

[48] ZFIN Publication: Westerfield, 1995. https://zfin.org/ZDB-PUB-970327-24.

[49] Terajima M., Taga Y., Cabral W. A., Nagasawa M., Sumida N., Hattori S., Marini J. C., Yamauchi M., Cyclophilin B Deficiency Causes Abnormal Dentin Collagen Matrix. J Proteome Res 16, 2914–2923 (2017). 28696707 10.1021/acs.jproteome.7b00190

[50] Teramura N., Tanaka K., Iijima K., Hayashida O., Suzuki K., Hattori S., Irie S., Cloning of a novel collagenase gene from the gram-negative bacterium Grimontia (Vibrio) hollisae 1706B and its efficient expression in Brevibacillus choshinensis. J Bacteriol 193, 3049–3056 (2011). 21515782 10.1128/JB.01528-10 PMC3133194

[51] Nagai N., Hosokawa M., Itohara S., Adachi E., Matsushita T., Hosokawa N., Nagata K., Embryonic lethality of molecular chaperone hsp47 knockout mice is associated with defects in collagen biosynthesis. J Cell Biol 150, 1499–1506 (2000). 10995453 10.1083/jcb.150.6.1499 PMC2150697

[52] Akaike T., Yoshida M., Miyamoto Y., Sato K., Kohno M., Sasamoto K., Miyazaki K., Ueda S., Maeda H., Antagonistic action of imidazolineoxyl N-oxides against endothelium-derived relaxing factor/.NO through a radical reaction. Biochemistry 32, 827–832 (1993). 8422387 10.1021/bi00054a013

[53] Kagiyama S., Tsuchihashi T., Abe I., Fujishima M., Cardiovascular effects of nitric oxide in the rostral ventrolateral medulla of rats. Brain Res 757, 155–158 (1997). 9200511 10.1016/s0006-8993(97)00336-3

