# Supplementary figures and images for: A facile method for fluorescent visualization of newly synthesized fibrous collagen by capturing the allysine aldehyde groups serving as cross-link precursors
Source: bioRxiv. 2025 Jun 24:2025.06.19.660320. Preprint. [Version 1] doi: 10.1101/2025.06.19.660320 (PMC12262523; doi:10.1101/2025.06.19.660320)

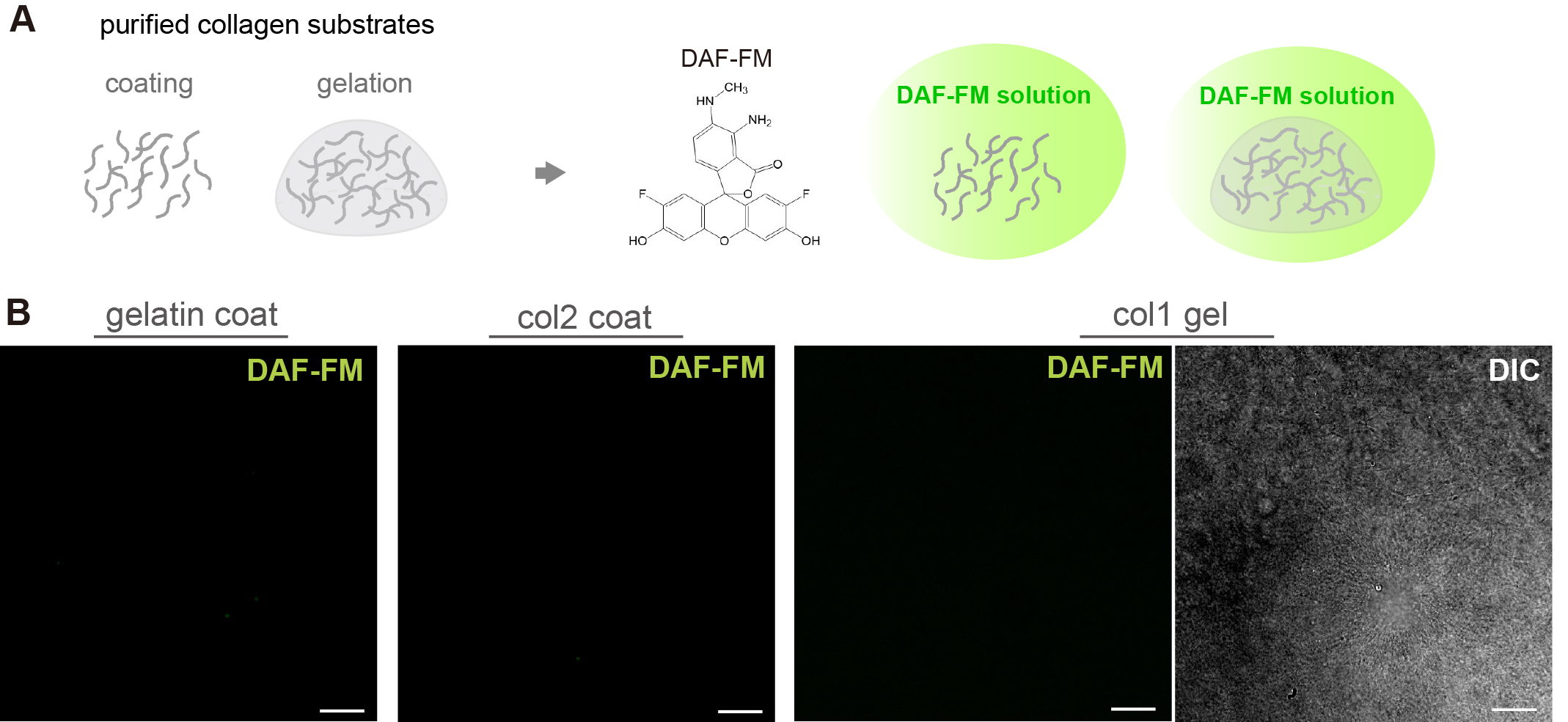

Supplement: Supplement 1 — fig. S1. Reactivity of DAF-FM with purified collagen. (A) Schematic diagram of DAF-FM staining for the purified collagen substrates. (B) Representative confocal images of the purified collagen after DAF-FM staining. Scale bar = 50 μm. [file media-1.jpg]

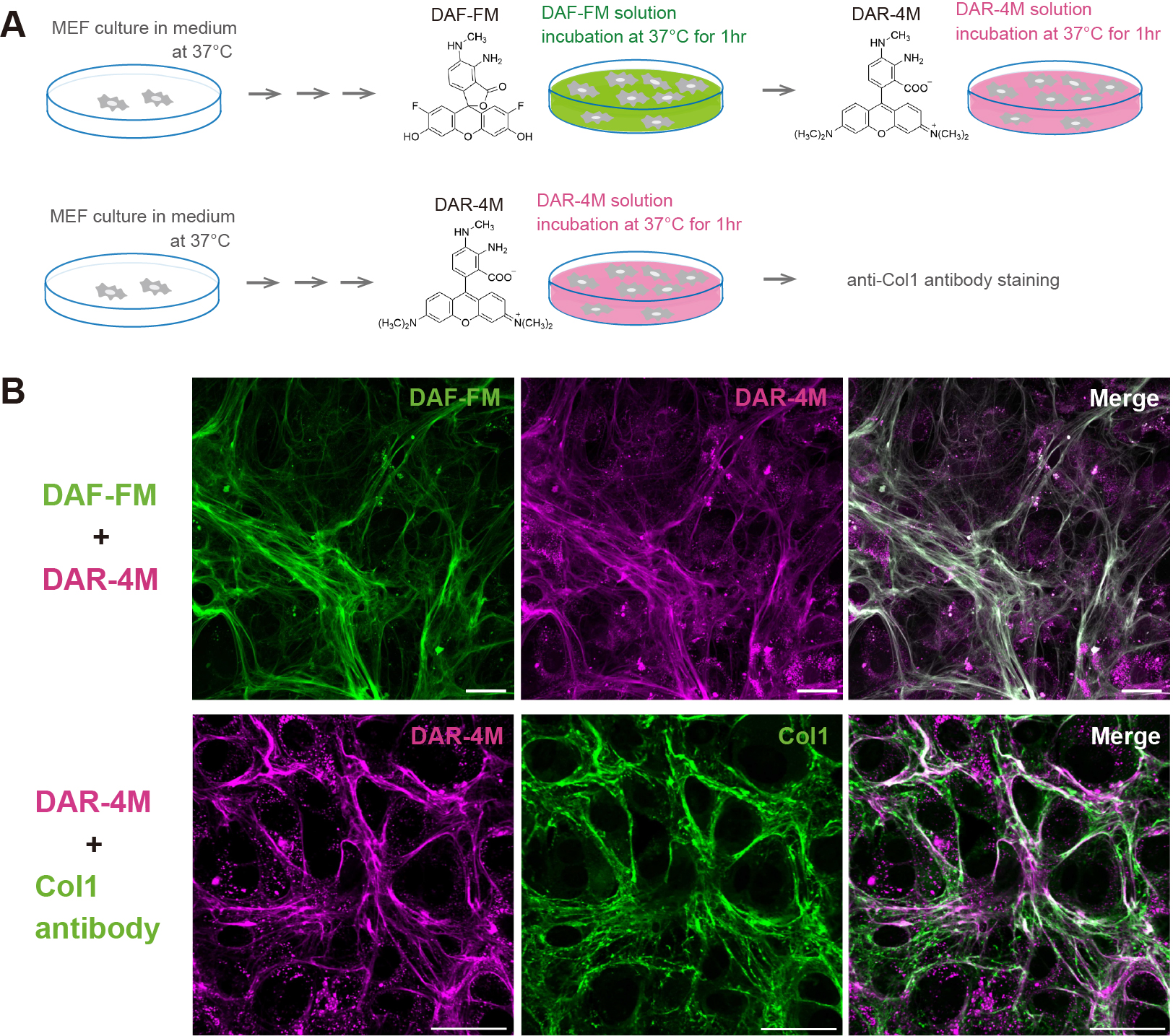

Supplement: Supplement 2 — fig. S2. Fluorescent staining of the collagen fibers formed by culture cells using DAR-4M. (A) Schematic diagram of DAR-4M staining for the collagen fibers formed by MEFs. (B) Upper panels show representative fluorescent images of the collagen fibers visualized with DAF-FM (green) and DAR-4M (magenta) at culture day 10. Both probes visualized the same fibers. Lower panels show representative fluorescent images of the collagen fibers visualized with DAR-4M (magenta) and anti-Col1 antibody staining at culture day 10. The fluorescent signals of DAR-4M and anti-Col1 antibody staining were merged well on the same fibers. Scale bar = 50 μm. [file media-2.jpg]

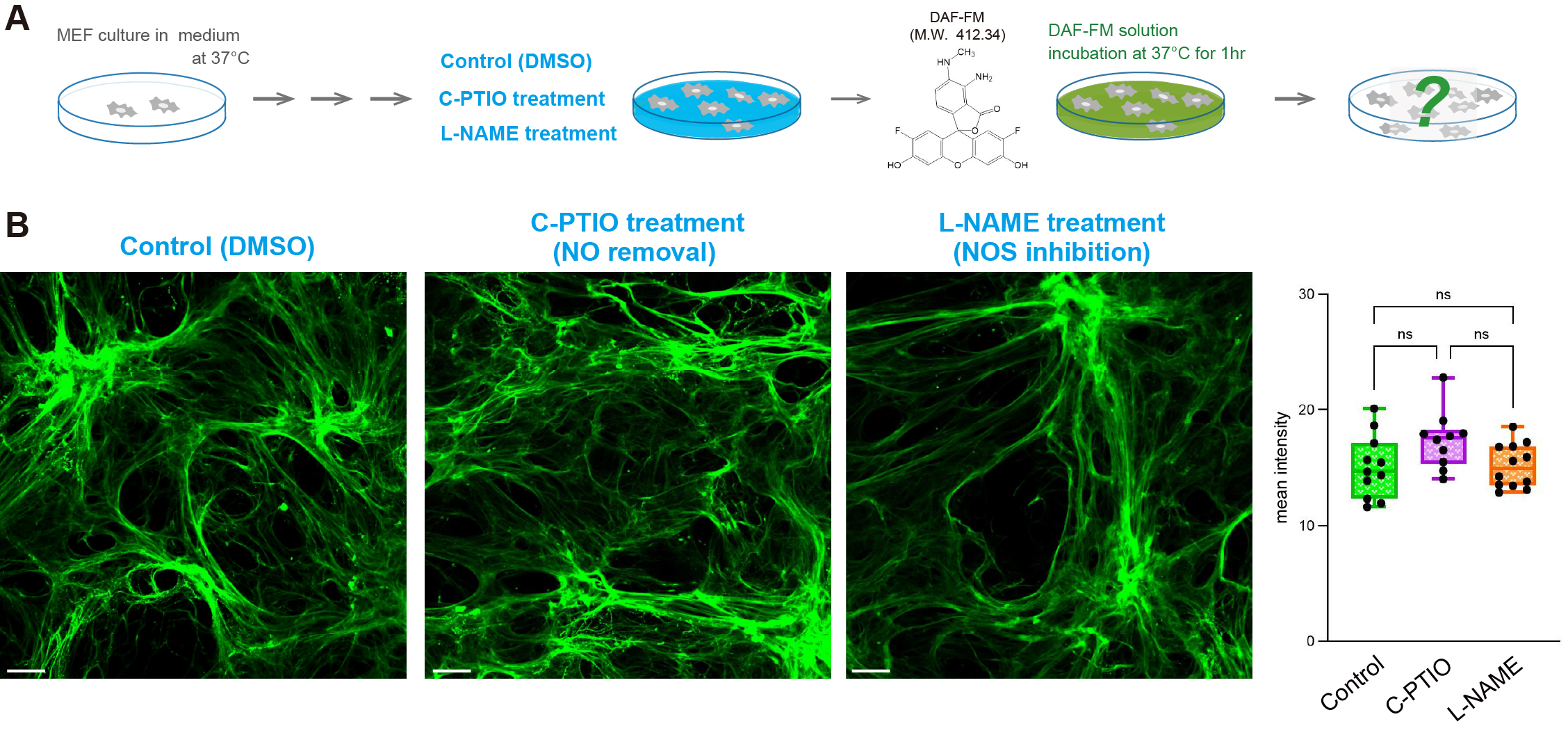

Supplement: Supplement 3 — fig. S3. NO-independent fluorescent visualization of collagen fibers by DAF-FM. (A) Schematic diagram of DAF-FM staining under NO removal conditions for the collagen fibers formed by MEFs. (B) Representative fluorescent images of the collagen fibers visualized with DAF-FM (green) at culture day 10. Clear fluorescent signals of collagen fibers stained with DAF-FM were detected under C-PTIO (NO removal) and L-NAME (NOS inhibition) treatment conditions, similar to the control. The fluorescence intensity plots for each condition are shown in the right panel. Scale bar = 50 μm. [file media-3.jpg]

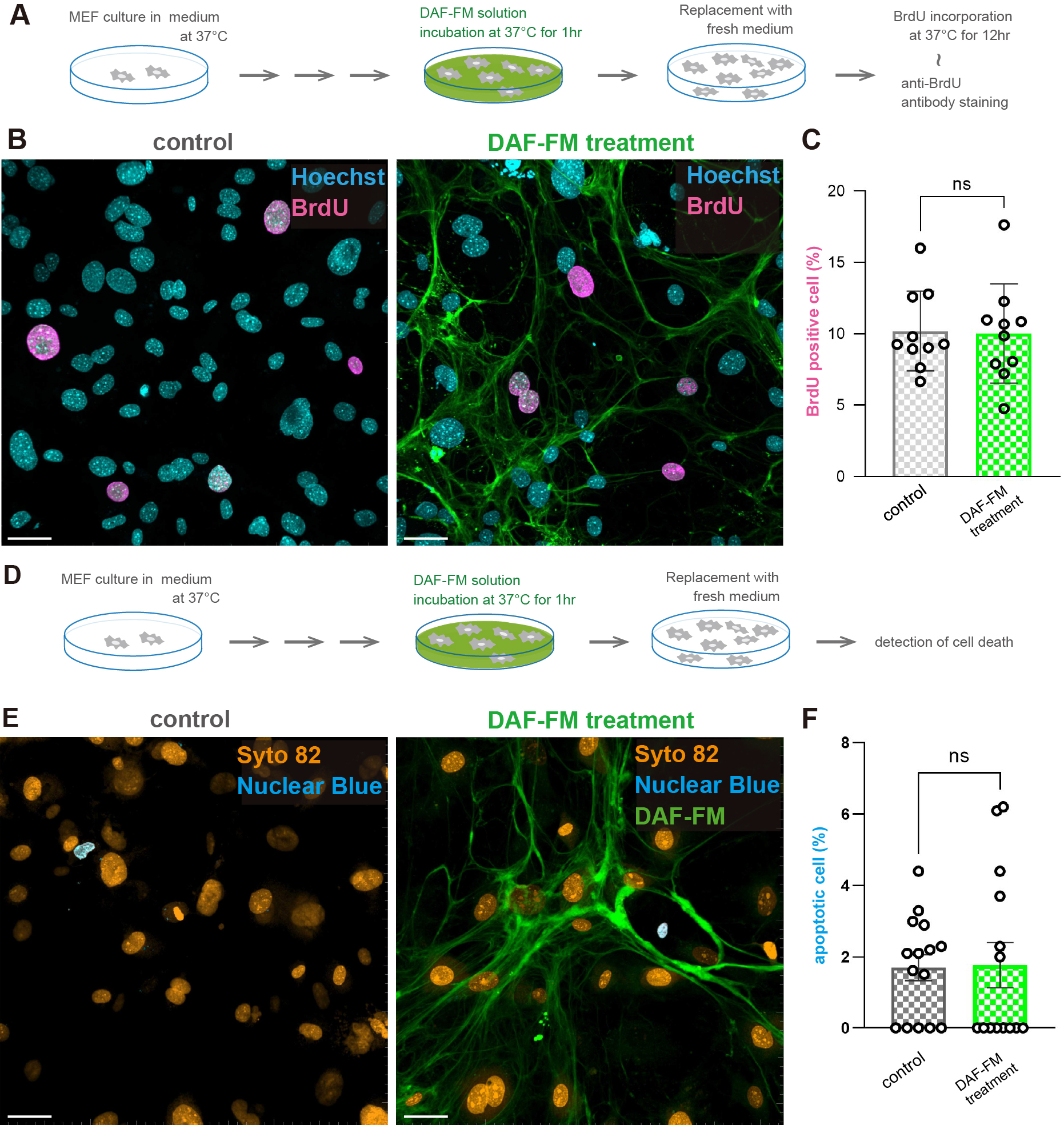

Supplement: Supplement 4 — fig. S4. No negative effects on cell activities with DAF-FM treatment. (A) Experimental workflow to investigate the effect of DAF-FM treatment on cell division. (B) Representative images of anti-BrdU antibody staining. Cultured MEFs were incubated with DMSO (control) or DAF-FM solution. All cell nuclei were stained with Hoechst (blue), and cell nuclei incorporating BrdU were stained with anti-BrdU antibody (magenta). Collagen fibers were stained with DAF-FM (green). (C) Number of BrdU-positive cells was counted under each condition. There was no significant difference in the percentage of BrdU-positive cells between the conditions. (D) Experimental workflow to investigate the effect of DAF-FM treatment on cell death. (E) Representative images of Nuclear Blue staining. Cultured MEFs were incubated with DMSO (control) or DAF-FM solution. All cell nuclei were stained with Syto 82 (orange), and cell nuclei of apoptotic cells were stained with Nuclear Blue (blue). Collagen fibers were stained with DAF-FM (green). (F) Number of apoptotic cells was counted under each condition. There was no significant difference in the percentage of apoptotic cells between the conditions. Scale bar = 50 μm. [file media-4.jpg]

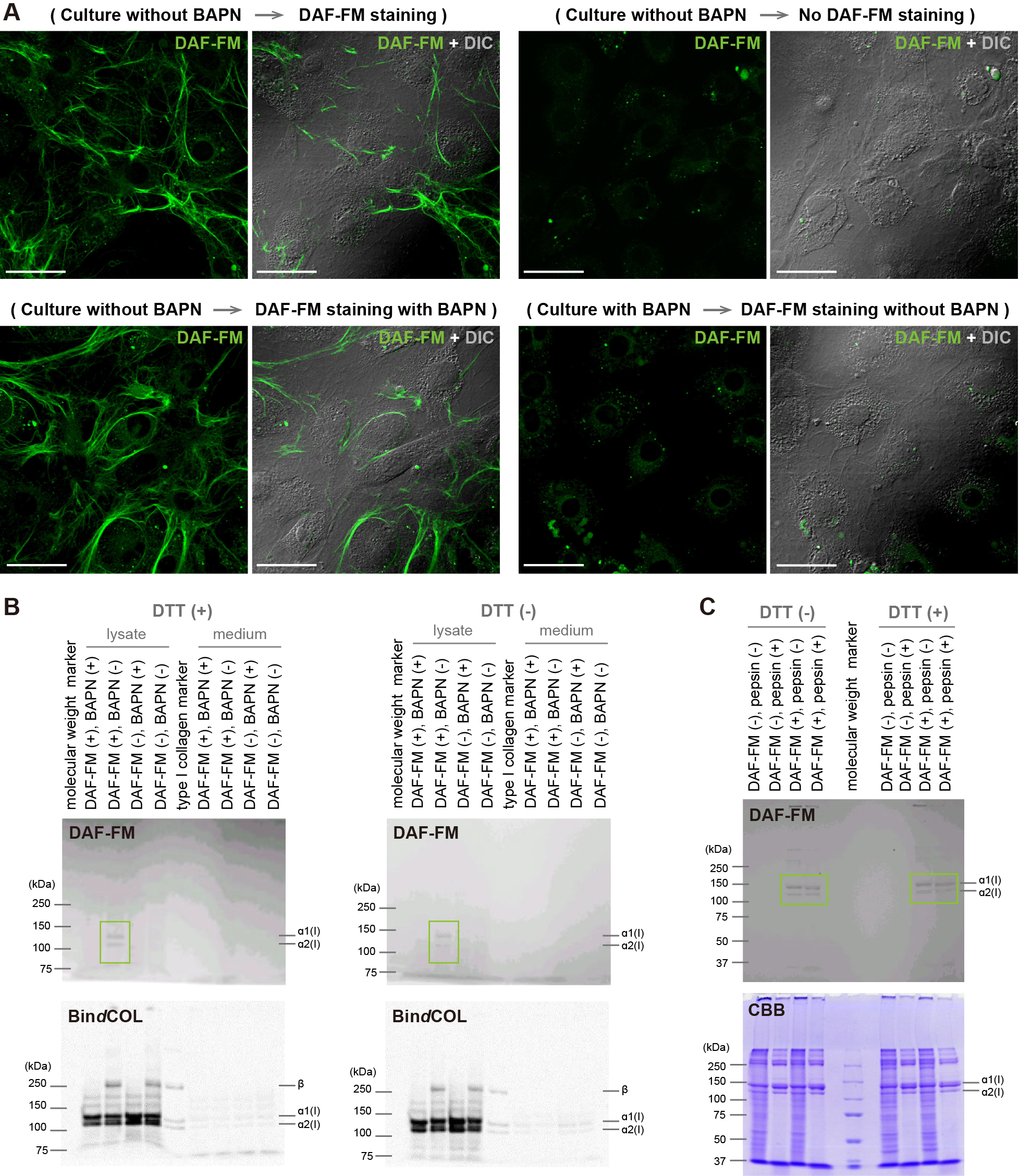

Supplement: Supplement 5 — fig. S5. DAF-FM fluorescence suppressed by inhibition of collagen cross-linking formation (A) Representative fluorescent images of the collagen fibers labeled by DAF-FM (green) under the various culture conditions. DAF-FM fluorescence of the collagen fibers produced by MEFs is not suppressed when BAPN is present only during DAF-FM staining, but it is significantly suppressed when BAPN is present during MEF culture. Scale bar = 50 μm. (B) SDS-PAGE analysis of DAF-FM-labeled collagen with or without DTT. DAF-FM fluorescence of proteins produced by MEFs in the presence or absence of BAPN was examined (upper panel), and BindCOL staining of the same protein samples was performed (lower panel). (C) DAF-FM fluorescence of pepsin-digested or undigested proteins produced by MEFs was examined (upper panel), and CBB staining of the same protein samples was performed (lower panel). [file media-5.jpg]

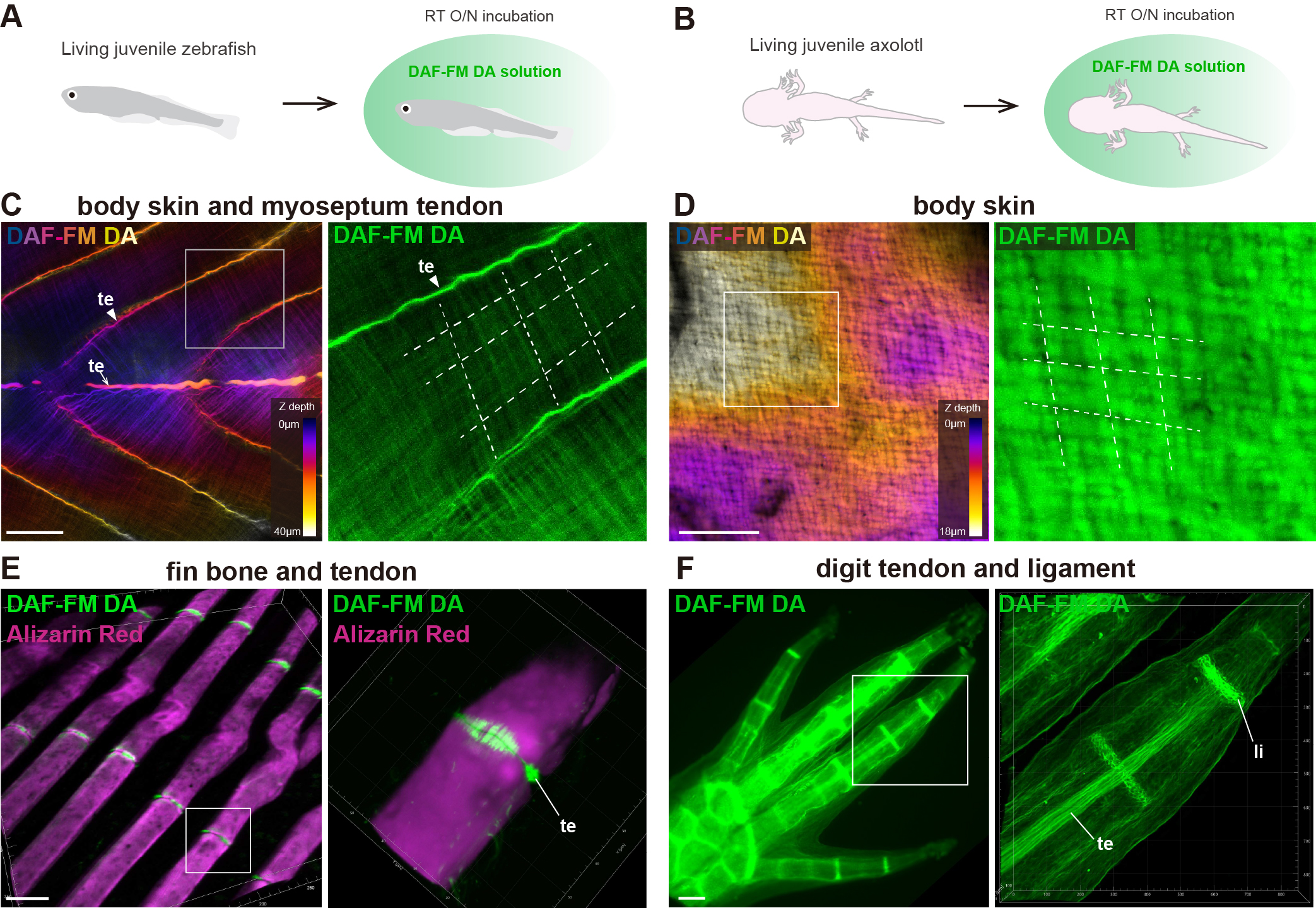

Supplement: Supplement 6 — fig. S6. Fluorescent visualization of collagen fibers in zebrafish and axolotl using DAF-FM DA. (A, B) Schematic diagram of DAF-FM DA staining for the collagen fibers in zebrafish and axolotl. Living juvenile zebrafish and axolotl were incubated overnight in 10μM DAF-FM solution. After the incubation, DAF-FM DA fluorescence in the animal tissues were imaged by a confocal microscopy. (C, D) Representative fluorescent image with depth color-coded MIP of the body skin labeled by DAF-FM DA in zebrafish and axolotl, respectively. The magnified MIPs of the areas within the white boxes are shown in the right panels of each image. White dotted lines indicate the orientation of collagen fibers. (E) Representative fluorescent images of the tendon labeled by DAF-FM (green) in the zebrafish fin bones. Fin bones were stained with Alizarin Red (magenta). (F) Representative fluorescent images of the tendon and ligament labeled by DAF-FM DA (green) in the axolotl forelimb digits. te, tendon; li, ligament. Scale bar = 50 μm (B, C and F) and 200 μm (E). [file media-6.jpg]

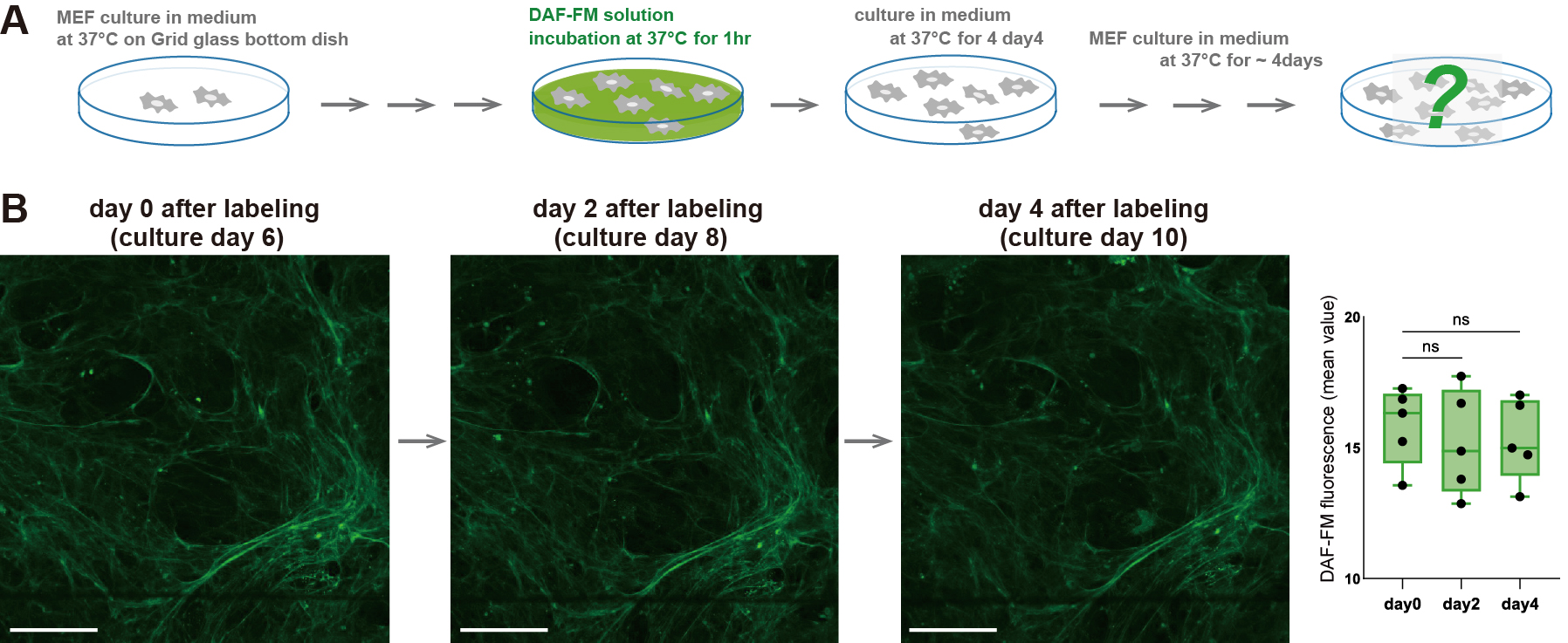

Supplement: Supplement 7 — fig. S7. Fluorescence of the collagen fibers labeled with DAF-FM hardly fade after washout. (A) Schematic diagram of the temporal observation after DAF-FM staining for the collagen fibers formed by MEFs. (B) Representative fluorescent images of the collagen fibers labeled with DAF-FM at day 0, day 2 and day4 after labeling. Fluorescent mean intensity values of the DAF-FM at each time point are shown in the right panel. Scale bar = 100 μm. [file media-7.jpg]

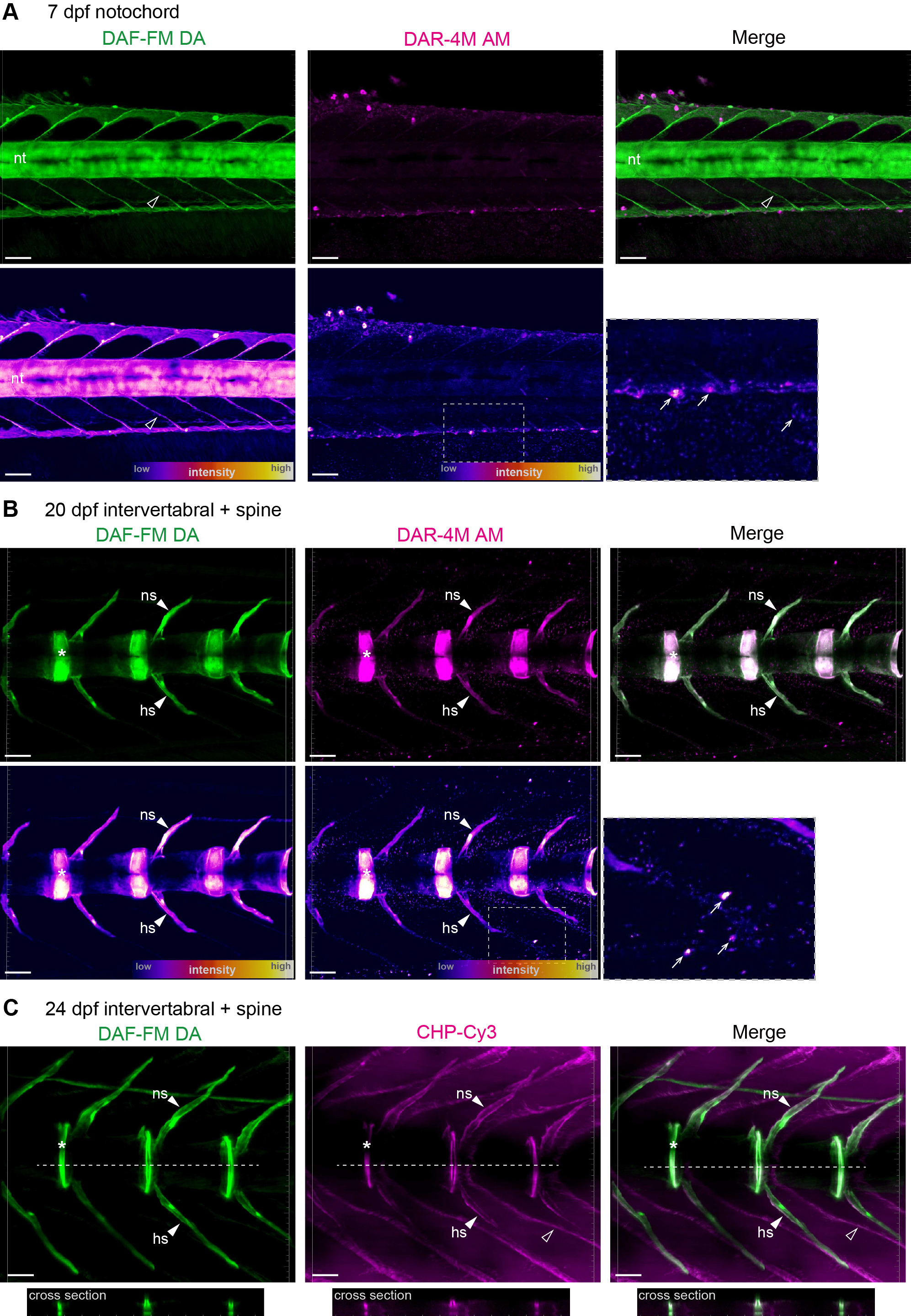

Supplement: Supplement 8 — fig. S8. Reactivity of DAF-FM DA and DAR-4M AM with zebrafish notochord and vertebral bones. (A) Representative confocal images of the collagen fibers in the notochord at 7 dpf simultaneously labeled with DAF-FM DA and DAR-4M AM. Each image with fluorescence intensity color-coded MIPs is shown in the lower panels. (B) Representative confocal images of the collagen fibers in the intervertebral regions and spines at 20 dpf simultaneously labeled with DAF-FM DA and DAR-4M AM. Each image with fluorescence intensity color-coded MIPs is shown in the lower panels. (C) Representative confocal images of the collagen fibers in the intervertebral regions and spines at 24 dpf co-labeled with DAF-FM DA and CHP-Cy3. Cross section images at the position of the white dotted lines in each fluorescent image are shown in the lower panels. nt, notochord; ns, neural spine; hs, hemal spine. Open arrowheads indicate the myoseptum tendon, arrows indicate unknown nonspecific signals detected in DAR-4M AM labeled samples, and asterisks indicate the intervertebral discs. Scale bar = 50 μm. [file media-8.jpg]
